# Fast Charging and High Power Ionogel‐Based Rain Energy Harvester

**DOI:** 10.1002/advs.202504608

**Published:** 2025-06-30

**Authors:** Ruoxuan Ye, Irum Firdous, Muhammad Fahim, Jihong Shi, Weilu Li, Xiangkun Bo, Fei Lui, Walid A. Daoud

**Affiliations:** ^1^ Department of Mechanical Engineering City University of Hong Kong Hong Kong 999077 China; ^2^ Shenzhen Research Institute City University of Hong Kong Shenzhen 518000 China

**Keywords:** droplet energy harvesting, ionogel, rain, steam sensing, triboelectrification

## Abstract

The growing demand for sustainable energy has triggered the exploration of innovative solutions to harness clean and renewable sources. In this pursuit, harvesting rain energy has emerged as a promising approach due to its unlimited availability. However, traditional droplet‐based harvesters face several challenges, including a long charging period, low electrical power output, and fast charge dissipation. This study presents an innovative ionogel‐based rain energy harvester that overcomes these limitations. The key advancement lies in the stable polarized interface created by the ionogel, which enables rapid charging and high charge retention, leading to a significant instantaneous power within the first few raindrops and achieving an 8‐fold increase in saturation performance over conventional design. The harvester only requires 25 droplets to reach saturation and maximum electrical output, achieving an impressive power density of 235.11 W m^−2^ and a conversion efficiency of 6.77%, being the highest recorded for ionogel and hydrogel‐based droplet energy harvesters. Additionally, the adhesive nature of the ionogel enhances the device's stability and versatility in varying energy generation and steam leakage applications. This work advances the pursuit of sustainable energy harvesting by bridging materials science with practical application.

## Graphical abstract

This work presents a highly adhesive ionogel‐based rain energy harvester, which enhances energy harvesting stability and efficiency (6.77%). This device is flexible, transparent, and exhibits exceptional electrical outputs, generating 502 µA and 204 V with every individual tap water droplet, while also showing promise in droplet energy harvesting and steam sensing applications.

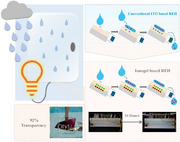

## 1 Introduction

In an era characterized by high energy consumption demands, the energy crisis remains a significant challenge. Global warming and extreme weather, driven by greenhouse gas emissions from traditional energy resources, also underscore the urgent need for a sustainable energy solution.^[^ ^1^ ^]^ Sustainable energy is renewable, environmentally friendly, and not dependent on fossil fuels.^[^ ^2^, ^3^, ^4^ ^]^ Among various clean energy sources, water holds great potential due to its vast global presence, covering more than 70% of Earth's surface. However, the current utilization of hydropower from rivers and oceans remains insufficient. Although the share of hydropower in total energy production has grown annually, it still does not exceed 7%.^[^ ^5^ ^]^ This highlights the necessity to explore additional forms of hydropower.^[^ ^6^ ^]^ Efforts to harvest droplet energy based on the coupling of triboelectrification and electrostatic induction have shown promise.^[^ ^7^, ^8^, ^9^, ^10^ ^]^ However, the energy harvesting efficiency remains low due to the properties of rain droplets, such as their low weight and frequency.

The droplet‐based electricity generator (DEG), introduced in 2020, has significantly addressed a key challenge by converting the conventional interfacial effect into a bulk effect, thereby increasing instantaneous power density by several orders of magnitude.^[^ ^11^ ^]^ Once saturated, it can illuminate hundreds of LEDs with just a single drop of water. This unprecedentedly efficient droplet energy harvester has sparked significant interest, leading to a series of mechanism studies^[^ ^12^, ^13^, ^14^, ^15^ ^]^ and optimizations, based on its simple three‐layer structure, which consists of a top electrode, a tribonegative layer, and a bottom electrode.^[^ ^16^, ^17^, ^18^, ^19^ ^]^ To further enhance energy harvesting, combinations of DEG with other forms of devices have emerged, boosting energy harvesting efficiency and serving additional purposes. For example, a DEG‐piezoelectric hybridized nanogenerator has been developed for droplet mechanical energy harvesting.^[^ ^20^ ^]^ Additionally, incorporating a water triboelectric nanogenerator on an amphiphilic cactus surface allows for the simultaneous harvesting of both the water and droplet energy.^[^ ^21^ ^]^ The integrated solar panel with a highly transparent triboelectric nanogenerator can also increase power output.^[^ ^22^, ^23^ ^]^

However, the prolonged charging process presents a challenge. As demonstrated, DEG requires 16 000 droplets to reach saturation and attain the maximum charge density, which is critical for energy efficiency. In practice, rain is often unpredictable and may not persist long enough to ensure optimal charging. If the droplet energy harvester necessitates such a substantial volume of rain, it becomes impossible to consistently maximize output. To address this problem, a switched effect based on an electric‐double‐layer capacitor has been introduced, utilizing an equivalent circuit model, where the charges stored contribute to a high‐power output from a single droplet without the need for pre‐charging.^[^ ^24^ ^]^ Additionally, both charge storage and charging time in the tribonegative layer can be controlled by systematically modeling and optimizing the hydrodynamics and circuit systems.^[^ ^25^ ^]^ Furthermore, the ionogel induction layer facilitates interfacial polarization with the tribonegative layer, which has the potential to accelerate the charging process and retain charges on the surface. However, the role of ionogel in the interfacial charging state of droplet energy harvesters has not yet been explored.

In this study, we designed a three‐layer rain energy harvester (REH), composed of aluminum (Al), fluorinated ethylene propylene (FEP), and an ionogel. The design can achieve high instantaneous power output in a short charging time, while also offering the advantages of high transparency and stability. The excellent adhesion properties of the ionogel enable it to maintain structural integrity under significant tension, making it suitable for applications in harsh and complex natural environments. Notably, the closely fitted FEP and ionogel create a stable polarized layer between the conductive layer and the tribonegative layer. This interface is established through covalent, ionic, hydrogen bonding, and lone pair electrostatic interactions that contribute to a high charge delocalization density.^[^ ^26^, ^27^ ^]^ This polarized interface effectively addresses issues such as prolonged charging time and low power output. Furthermore, this active layer not only enhances the field of droplet energy harvesting but is also recognized for its potential applications in flexible sensors for signal detection, super‐capacitors for power reserve, and smart interfaces. Additionally, we explored its potential in steam leakage sensing.

## 2 Results and Discussion

The cornerstone of the ionogel‐based rain energy harvester (i‐REH) lies in its fundamental departure from conventional saturation‐dependent systems. Unlike traditional ITO/metal film‐based devices, which require extensive pre‐charging cycles (e.g., 16 000 droplets to reach saturation^[^ ^11^ ^]^), i‐REH leverages interfacial polarization between the FEP tribonegative layer and ionogel electrode, establishing an intrinsic charge reservoir. This polarized interface, stabilized by covalent, ionic, and hydrogen bonding interactions, eliminates the requirement for droplet‐driven charge accumulation. FTIR and UV–vis analysis confirm that the sulfate‐rich network facilitates anion migration to FEP, creating a pre‐polarized electric double layer (EDL). Consequently, the system achieves functional output with only 25 droplets (vs 150 droplets for the ITO‐based counterpart) and maintains stability across intermittent rainfall cycles (10 min with < 1.5% charge loss).

### 2.1 Material Properties

**Figure 1:**
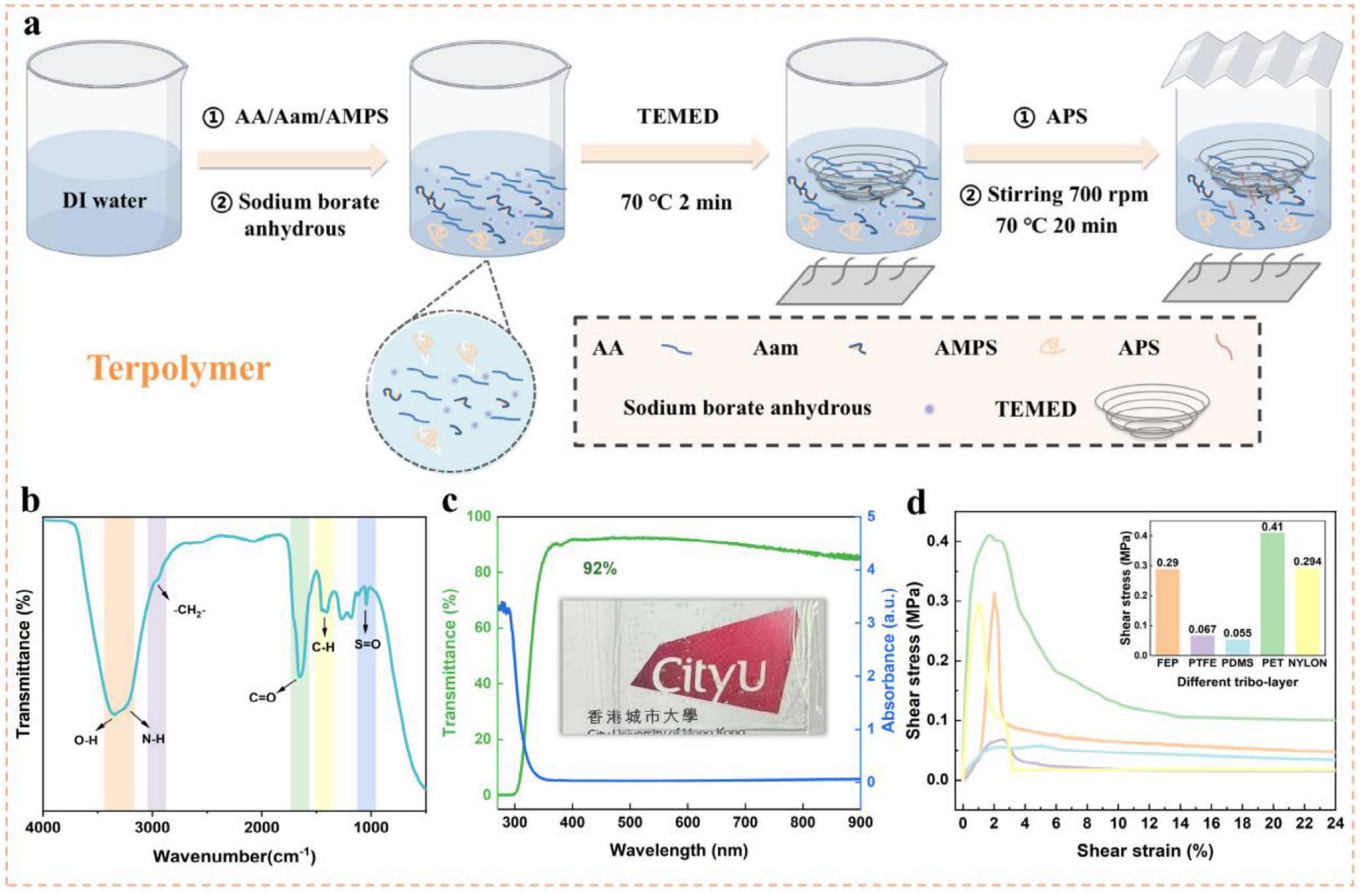
Polymerization of the terpolymer ionogel and demonstration of its structure, bonding, and adhesive properties. a) Synthesis process of terpolymer ionogel. b) FTIR spectra and c) UV–vis transmittance and absorbance spectra. Mechanical adhesion test of five different films: d) Shear stress versus shear strain.

The terpolymer ionogel presented herein is synthesized through a meticulous free‐radical polymerization process. A precise blend of three distinct monomers, acrylic acid (AA), acrylamide (Aam), and 2‐acrylamido‐2‐methyl‐1‐propanesulfonic acid (AMPS), is homogeneously dispersed in deionized water alongside the cross‐linker sodium borate anhydrous. Subsequently, tetramethyl ethylenediamine (TEMED) is introduced as a potent accelerator, enhancing the polymerization kinetics. Ammonium persulfate (APS) is then swiftly incorporated as the initiator. This facile one‐pot strategy yielded the target terpolymer ionogel material in a green and scalable manner (**Figure** **1**a), and its chemical components are illustrated in Figure S1 (Supporting Information). They contribute to the hydrophobicity of this ionogel (Figure S2, Supporting Information).

Figure 1b (FTIR) affirms that the covalent/ionic bonding is in the terpolymer. The ionogel exhibits > 92% transmittance in the visible range (Figure 1c), enabling direct integration with solar panels or windows without obstructing light access. A comprehensive discussion is presented in Note S1 (Supporting Information).

The ionogel is crucial in forming a solid device structure. To evaluate its mechanical properties, the adhesive tests were conducted in a sandwich structure comprising a glass substrate, the ionogel, and films of 5 different materials, FEP, polytetrafluoroethylene (PTFE), polydimethylsiloxane (PDMS), polyethylene terephthalate (PET), and nylon, as illustrated in Figure S3 (Supporting Information). The reason these films were selected was not only that they are classical tribo‐materials, which have different triboelectric polarities, but also due to the versatility of ionogel with varying surface properties, such as surface energy, roughness, and chemical composition.

The results shown in Figure 1d indicate that PET has the largest shear stress of 0.41 MPa, which is 7‐fold greater than PDMS. The large shear stresses can be tolerated by FEP and nylon, which are higher than 0.29 MPa. This is attributed to enhanced interfacial bonding that results from their higher surface energy compared to PDMS and PTFE. Additionally, PET, FEP, and nylon are plastic and have flat surfaces, and are thus more conducive to adhesion than PDMS and PTEF, which are softer and rougher.

On balance, FEP was selected as the tribonegative layer. FEP not only has a relatively high adhesion compatibility with the ionogel, but also higher electron affinity than PET. Therefore, the high mechanical strength contributed by the ionogel is a crucial factor toward practical application, as it makes the device more robust, and the high electron affinity facilitates higher charge generation. Moreover, the robust adhesion between ionogel and FEP layers plays a crucial role in maintaining the stability of the inherent EDL at their interface. The strong interfacial bonding ensures continuous anion migration from ionogel to FEP, as confirmed by FTIR analysis. Further, the tight adhesion prevents delamination or microcap formation that could disrupt the EDL, effectively acting as a charge confinement barrier.

### 2.2 Working Schematic and Output Performance Including Charge Stability

**Figure 2:**
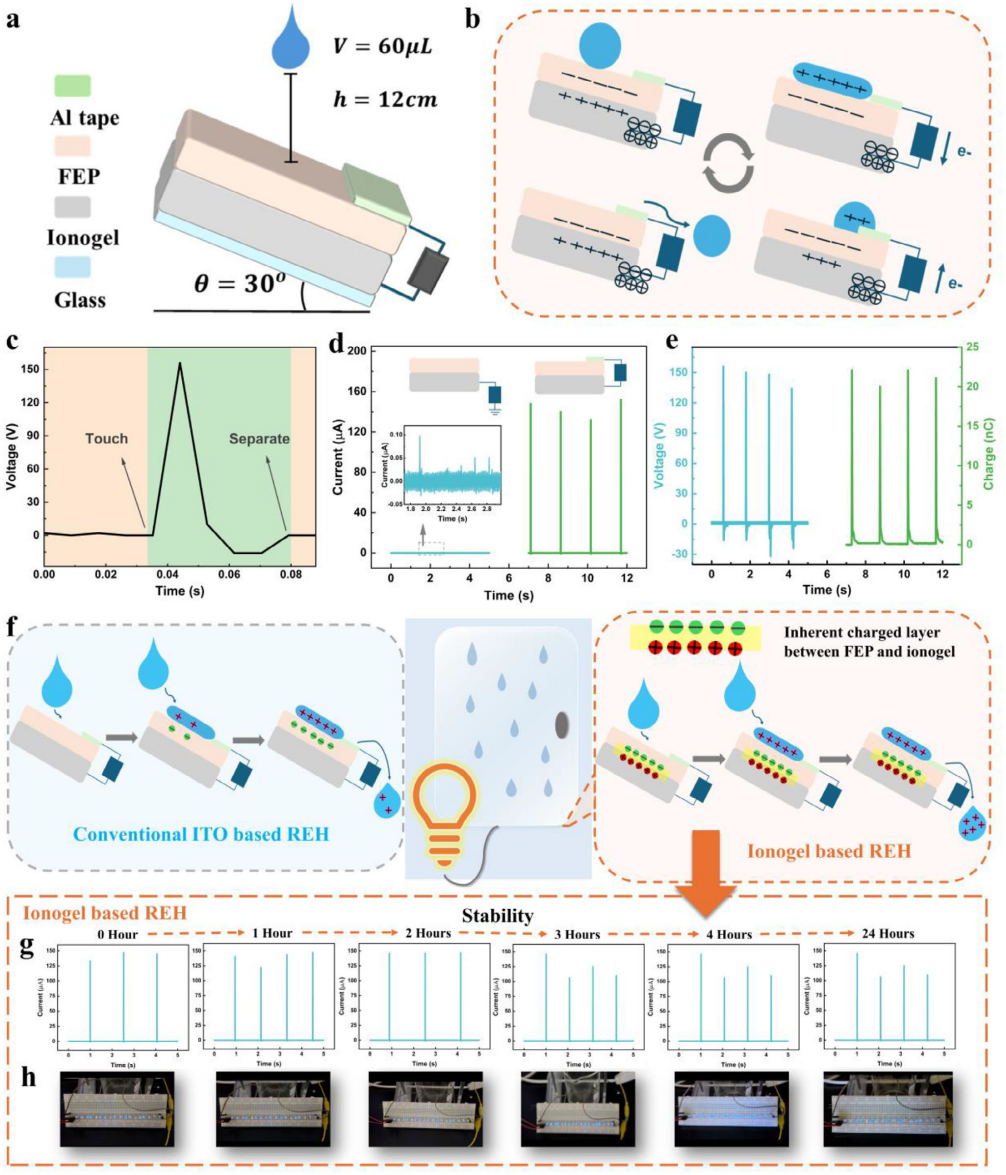
Working schematic and performance of i‐REH. a) Schematic diagram of i‐REH. b) Working mechanism of i‐REH with a whole working cycle. c) One cycle of voltage output. d) Short circuit current comparison of i‐REH with a single‐electrode droplet energy harvester. e) Outputs of voltage and charge of i‐REH. f) Comparison of a conventional ITO‐based REH with gradual charging (blue) and ionogel‐based i‐REH with an inherent charged layer (orange). g,h) Charge stability assessment of i‐REH.

In order to prove the validity of the i‐REH made of ionogel, its working schematic and performance were investigated. **Figure** **2**a presents a schematic illustration of the device, comprising three primary components: an FEP tribonegative layer, chosen for its transparency, hydrophobicity, strong adhesion to the ionogel, and electron affinity. The ionogel serves as the bottom electrode, tightly bonding the FEP film to the glass substrate, thereby forming a robust structure. A small piece of Al tape is placed on the FEP surface to function as the top electrode, as depicted in Figure S4 (Supporting Information). To ensure consistency of the experimental results, i‐REH is fixed at a constant inclination angle of 30°. Water droplets with a volume of 60 µL are released from a height of 12 cm at a frequency of 1.2 droplets per second, and the droplet simulation setup is shown in Figure S5 (Supporting Information).

To explain the working mechanism of i‐REH, Figure S6 (Supporting Information) demonstrates the equivalent capacitance circuit diagram of this device.^[^ ^11^ ^]^ In addition to the capacitance between FEP and ionogel C_F/I_, several other capacitances are present: the capacitance of FEP C_F_, the EDL of C_A/D,_ formed at the interface between the droplet and the top electrode, and C_D/F,_ formed between the droplet and FEP.^[^ ^28^ ^]^ Additionally, there is a capacitor representing the EDL between the ionogel and the metal wire, C_I_.^[^ ^29^, ^30^ ^]^ When the droplets are not in contact with either the top electrode or FEP, the circuit remains in a disconnection state, resulting in no detectable electrical signal. However, once the droplet descends and establishes contact, the circuit transitions to an activated state, resulting in charge transfer from C_F_ to other capacitances. This “switched on” effect enables an instantaneous high‐power output, as the charge stored in FEP can be transferred to the other capacitors within the circuit, rather than being immobilized due to interfacial issues.^[^ ^31^ ^]^

The working cycle illustrated in Figure 2b further elucidates the phenomenon. When the droplet contacts FEP, it becomes negatively charged due to contact electrification, resulting in the formation of EDLs between the droplet and FEP. Simultaneously, induced positive charges accumulate on the ionogel bottom electrode. With the spread of droplets on FEP, these charges increase in proportion to the expanding droplet area. Once the droplet contacts the top electrode, the circuit is activated. Consequently, the charges accumulated transfer through this circuit, resulting in electron flow from the top to the bottom electrode to reach potential equilibrium, thereby generating an instantaneous output signal. As the droplet shrinks and eventually detaches from the top electrode, electrons return to the top electrode.

This process aligns with the signal shown in Figure 2c, which illustrates one cycle of voltage output. After the droplet contacts the top electrode, an instantaneous positive signal is observed, followed by a negative signal corresponding to droplet shrinkage. Finally, the voltage returns to zero, indicating that the droplet has left the device. Figure 2d presents a comparison of the current output with a conventional single‐electrode droplet energy harvester, which relies on traditional electrification and electrostatic induction. Due to interfacial effects, the charges accumulated on the tribonegative layer cannot be fully utilized, and thus the maximum current output is smaller than 0.1 µA, which is ≈1500‐fold smaller than that of i‐REH. This highlights the bulk effect of i‐REH and emphasizes the advantages of the “switch on” effect. At a steady state, with the continuous release of droplets, the current from a single droplet reaches 158 µA, while the voltage and charge remain stable at 174 V and 22.5 nC, respectively (Figure 2e).

In this study, i‐REH does not require a prolonged charge accumulation process, where only a few droplets could saturate the tribonegative layer and generate high electrical output, eliminating the requirement for hundreds of pre‐charged droplets. Additionally, the charges stored on FEP can be maintained over an extended period. Figure 2f compares a conventional ITO‐based device with i‐REH. The device in the blue section represents a conventional REH utilizing ITO glass as the bottom electrode, which necessitates a prolonged charging process. Initially, both the solid surface and the droplet are uncharged, and charge generation occurs when droplets begin to contact FEP. Due to different charge affinities, the charge separation occurs, resulting in positively charged droplets and negatively charged FEP. As additional droplets slide across the surface, the charges on FEP gradually increase until the rate of charge generation equals the rate of charge dissipation, reaching a steady state. Typically, this process requires hundreds or thousands of raindrops.

In contrast, i‐REH features ionic roots and elements with distinct chemical properties. Specifically, the ionogel is abundant in sulfate ions, whereas FEP is rich in fluorine atoms. The disparity facilitates charge separation at the interface and the formation of EDL. The charge separation at the interface is further augmented by the migration and adsorption of anions from the ionogel to FEP. Currently, molecular dipoles at the interface align along the electric field, creating an ordered dipole layer, which amplifies the interfacial charge effect. Consequently, the inherent charge layer between FEP and ionogel layer significantly reduces the number of droplets required for charging. Results in Figure S7 (Supporting Information) suggest that the conventional ITO‐based REH requires over 150 droplets to saturate the FEP layer and achieve stable outputs, while i‐REH requires only 25 droplets. The stable capacitor charging speed of i‐REH, as shown in Figure S8 (Supporting Information), further demonstrates its faster charging process and higher charging retention compared to its conventional ITO‐based counterpart. The saturation performance is devised to quantitatively compare the charging performance of two i‐REHs, as expressed in Equation 1,

(1) $$Saturation\;\;performance=\frac{Q_{max}}{N_{90\%}}$$

where the Q_max_ is the maximum charge output, and the N_90%_ is the number of droplets required to achieve 90% saturation. The saturation performance of the ionogel and ITO‐based REH is ≈0.1 and 0.84 nC, respectively, representing an 8‐fold enhancement.

Moreover, the inherent charged layer enhances the charge stability of i‐REH. For ITO‐based REH, the charges on FEP quickly dissipate due to surface conduction, bulk conduction, and neutralizing reaction with air when droplets cease to fall. Thus, in practical applications involving intermittent rain, this device must be recharged to maintain a high‐power density output, which reduces the efficiency of energy harvesting. Conversely, this ionogel, as characterized by its ionic conductivity and electrochemical stability, can help maintain a more stable charge on FEP. On one hand, the inherent charged layer formed between the tightly bonded FEP and ionogel is more stable than in an air environment, where charge dissipation occurs more rapidly. On the other hand, the ionogel contains numerous ions and charge carriers, allowing it to replenish FEP during dissipation, thereby enhancing the overall charge stability. The adhesion quality also contributes to a low charge decay rate, significantly outperforming conventional ITO‐based REH. By preserving the integrity of the ion‐pair alignment at the interface, the strong adhesion enables the formation of a discrete Helmholtz‐like EDL, which is crucial for the rapid charging and high charge retention capabilities of i‐REH.

In Figure 2g, the device stability was studied by initially activating the droplet simulator and collecting a five‐s electrical signal. Immediately afterward, the droplet simulator was closed. After allowing the device to remain in ambient conditions for a fixed duration, the droplet simulator was reopened to collect another five‐s electrical signal. This procedure was repeated five times. Finally, at the end of 24 h, an additional electrical signal was collected to assess charge dissipation. Simultaneously, the LEDs performance in Figure 2h was recorded. All six current output signals over the 24 h exhibit high stability, indicating minimum surface charge dissipation. The immediate illumination of the LEDs also demonstrates that the output power density remains stable. To further investigate their surface charge decay, Kelvin probe force microscopy (KPFM) was utilized to measure the contact potential difference (CPD) after fully charging and a 10‐min charge decay period (Figure S9, Supporting Information). The ITO‐based REH exhibited a significantly faster charge decay of ≈16.6%, while the ionogel‐based REH maintained a stable CPD, with only a 1.48% reduction.

### 2.3 Output Performance

**Figure 3:**
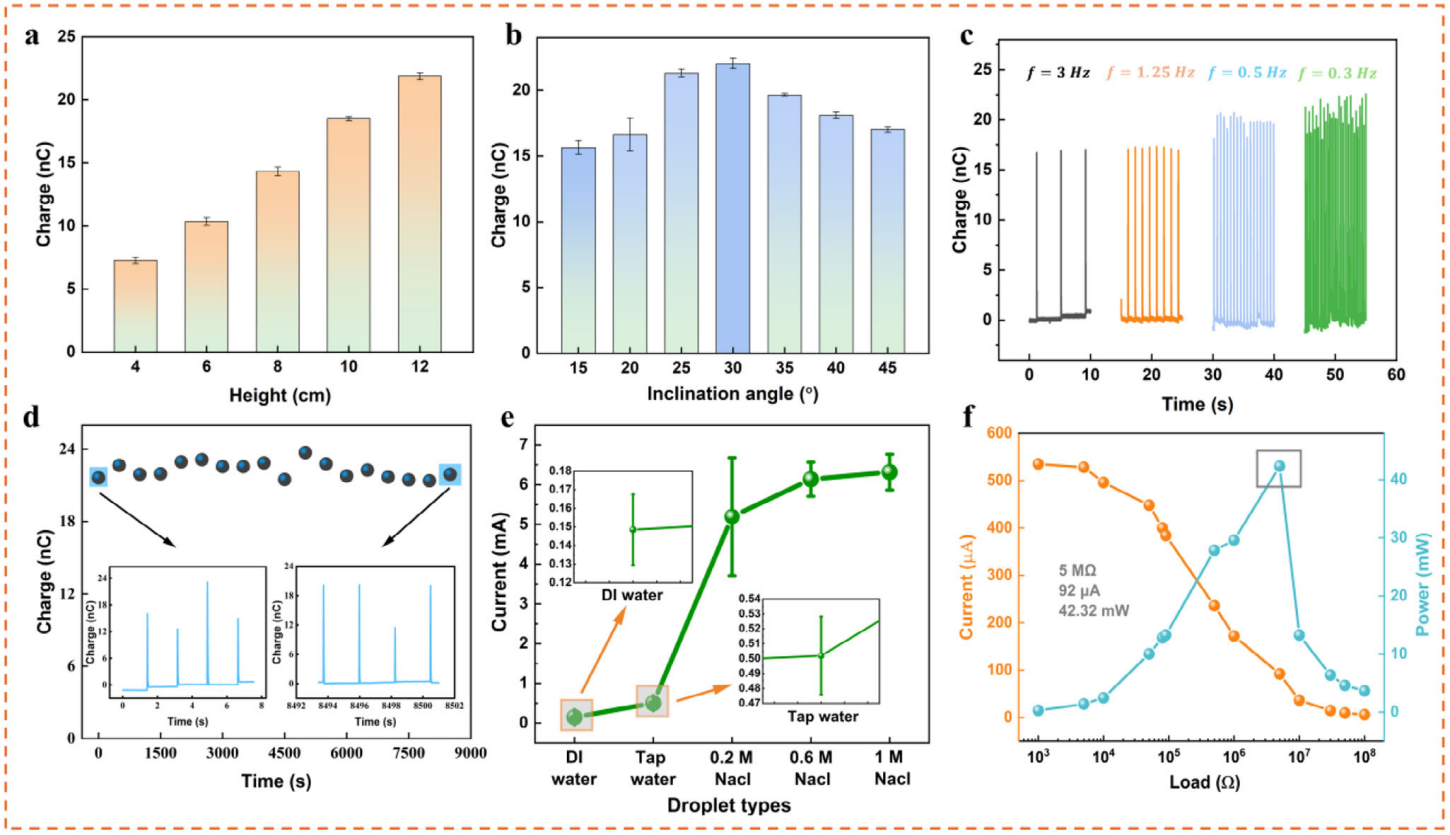
Output performance of i‐REH. Output performance study: a) Charge output with different droplet heights. Data are means ± the standard error of the mean (s.e.m). For each mean, the total number for measurement is 5. b) Charge output at different inclination angles. Data reported are means ± s.e.m. For each mean, the total number for measurement is 4. c) Charge output with different droplet frequencies. d) Electrical output durability for 8500 s under a frequency of 0.5 droplets per second. e) Output current of i‐REH under different droplet types of deionized water, tap water, and saline solutions of these concentrations (0.2, 0.6, and 1 m). Data reported are means ± s.e.m. For each mean, the total number for measurement is 5. f) Output current and power of i‐REH at different external load resistances under tap water.

To evaluate the performance of i‐REH in power generation, it is essential to simulate and optimize the output under varying conditions. First, the superior performance of the FEP and ionogel combination was confirmed by assessing the charge output of a number of bottom electrodes and tribo‐layers (Figure S10, Supporting Information). Subsequently, the outputs under varying droplet height, inclination angle, and droplet frequency were investigated. As shown in **Figure** **3**a, the output exhibits a strong correlation with droplet height. Specifically, the charge increased from 7.28 to 21.86 nC as the height increased from 4 cm to 12 cm, which can be explained by Equation 2 and the Weber number^[^ ^11^ ^]^

(2) $$Weber\;number=\rho Dv^{2}/\gamma$$

where ρ is the density, D is the diameter, υ is the impact velocity, and γ is the surface tension. A greater height not only increases the spread area of the droplet but also enhances the impact velocity upon contact with the tribonegative layer. Additionally, the increased impact pressure of the droplet accelerates electron transfer between the droplet and the tribonegative layer due to the overlap of the electron cloud.^[^ ^32^ ^]^ Consequently, these factors contribute to greater charge accumulation and transfer in the circuit.

Moreover, the charge output is significantly influenced by the inclination angle (Figure 3b). As the angle increased from 15° to 60° with an interval of 5°, the charge initially increased from 15.65 to 22.03 nC, with the maximum output occurring at 30° and then decreased to 17 nC. Increasing the inclination angle enhanced the droplet sliding velocity on FEP, resulting in accelerated electron transfer between the top and bottom electrodes. However, with the continuous angle increasing, the contact area of droplets with FEP diminishes, thus reducing the effective area of the EDL between droplet and FEP, leading to reduced charge transfer and output signal.

Subsequently, the relationship between droplet frequency and charge output was also investigated. This output shows less sensitivity to frequency compared to droplet height and inclination angle, as the spread area remains relatively constant (Figure 3c). However, a higher frequency can expedite the saturation of FEP, resulting in fewer opportunities for charge dissipation or neutralization, thereby increasing the charge output.^[^ ^16^ ^]^

Durability is critical for sustaining optimal power generation efficiency, particularly since rainfall events can last from several minutes to several hours. Therefore, continuous charge output monitoring with deionized water droplets for 8500 s was conducted. As illustrated in Figure 3d, the maximum charge output remains stable throughout this period, maintaining a value of ≈22 nC. The electrical signal plots for the initial 7.5 and final 7.5 s, shown in the two zoomed‐in subfigures, further substantiate the long‐term durability of i‐REH. Further, a 15‐day stability test of i‐REH has been carried out, where the voltage outputs remained stable with a small decrease occurring after 10 days (Figure S11, Supporting Information).

The current output is significantly influenced by the conductivity of the water droplets. Droplets with higher conductivity yield higher current output. In Figure 3e, the current generated by tap water droplets reaches ≈502 µA, which is more than three times greater than that of deionized water. Furthermore, the current outputs from NaCl solution droplets (0.2, 0.6, and 1 m) exceed 5 mA, representing a tenfold increase compared to that of tap water. In addition, the current and voltage output signals of tap water are shown in Figure S12 (Supporting Information), with values of 502 µA and 204 V, respectively.

Moreover, this i‐REH has demonstrated the capability to illuminate at least 125 LEDs, as shown in Figure S13 (Supporting Information), validating the high‐power density produced by a single droplet. To calculate the power density under tap water droplets, data of current and calculated power varying with increased external resistance are plotted in Figure 3f. At maximum power, the resistance of i‐REH is 5 MΩ, corresponding to a power output of 42.32 mW and a power density of 235.11 W m^−2^ (Assuming the maximum droplet spreading area is 1.8 cm^2^). Consequently, the energy conversion efficiency was calculated to be 6.77%, as detailed in Note S2 (Supporting Information). Both power density and conversion efficiency surpass many previously represented works (Table S1, Supporting Information). And its power output is the best among reported ionogel/hydrogel‐based i‐REH (Table S2, Supporting Information).

### 2.4 Practical Applications

The device presents a viable power supply solution during rainy days. The numerous advantages, including high electrical outputs, fast charging process, and high charge retention, enable its application across various scenarios. Notably, i‐REH can be easily assembled by applying the ionogel and placing the FEP and top electrode on any insulated substrate. The mechanical study indicated that the ionogel provides sufficient shear strength to ensure structural integrity, making it suitable for use in high‐speed vehicles. Additionally, the transparency of both the ionogel and FEP facilitates integration with solar panels or placement on building windows, enabling large‐scale rain energy harvesting. Furthermore, benefiting from its softness and weightlessness, i‐REH can conform to various curved surfaces, such as leaves, hat brims, and umbrellas. Three application scenarios were explored: charging a capacitor to power a stopwatch and integrating i‐REH with a PV panel and an umbrella.

**Figure 4:**
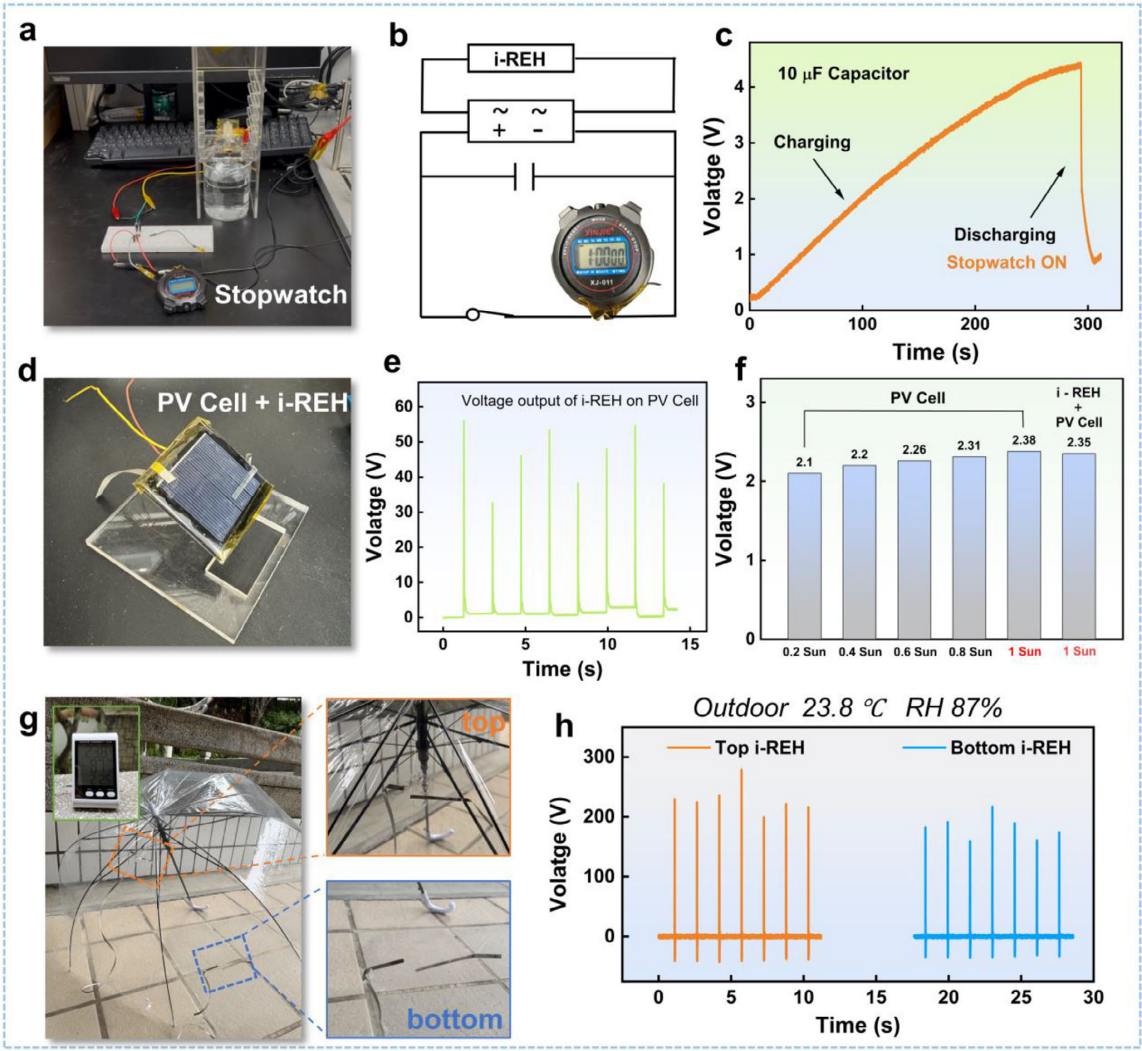
Application in droplet energy harvesting. a) Photo of the stopwatch power experiment. b) Equivalent circuit diagram. c) A 10 µF capacitor was charged to 4.4 V to power a stopwatch. d) Photo depicting the combination of i‐REH and photovoltaic (PV) cells. e) Voltage output of i‐REH on the PV cell with continuous deionized droplets. f) Open‐circuit voltage output of the PV cell under varying light intensities, including the combined system under 1 sun. g) Photo of two i‐REHs attached to an umbrella in an outdoor environment (temperature: 23.8 °C, humidity: 87%). h) Voltage output of two i‐REHs under tap water droplets.

**Figure** **4**a presents a photo of a stopwatch powered by i‐REH. The power generated by i‐REH is stored in a 10 µF capacitor via a rectifier, which is connected in parallel with a stopwatch (Figure 4b). Initially, the circuit is open, and the stopwatch is inactive. Once the voltage stored in the capacitor exceeds 4.4 V, the circuit closes, allowing the capacitor to power the stopwatch. As shown in Figure 4c, at a tap water droplet frequency of 0.5 droplets per second, the capacitor reaches 4.4 V in 4.6 min. When the circuit closes, the stopwatch turns on, and the capacitor's voltage gradually decreases until it is depleted.

Given that i‐REH exhibits outstanding transparency (greater than 92%), it was mounted on a PV cell, as shown in Figure 4d. This integrated system facilitates all‐weather energy generation. For example, the PV cell generates energy on sunny days, while i‐REH generates electricity on rainy days. Due to the strong adhesion properties of the ionogel, the fabrication process is straightforward, involving the application of an ionogel layer, followed by FEP, and a top electrode on top of the PV cell. Figure 4e demonstrates effective i‐REH operation, generating a stable voltage signal under continuous dropping of deionized water. Furthermore, Figure 4f displays the increasing output of the PV cell with increasing irradiance from 0.2 to 1 Sun (1 Sun typically refers to standard solar irradiance, which is ≈1000 W m^−2^). Notably, a comparison between the voltage output of the bare PV cell and that mounted with an i‐REH under 1 Sun shows a negligible impact of i‐REH on the performance of the PV cell, with a reduction below 1.26%.

The next application is particularly relevant to daily life as umbrellas are frequently and commonly used and can be employed to harvest energy from raindrops, as shown in Figure 4g. In order to validate the feasibility of i‐REH in an outdoor environment, this experiment was conducted entirely outdoors, with a temperature and humidity meter monitoring the conditions in real‐time. These i‐REHs on the umbrella operated stably, generating transient voltages exceeding 200 V (Figure 4h). The outputs followed the same trend observed in the previous test in Figure 3b, indicating that an inclination angle closer to 30° results in higher output. In addition, we explored the potential of ionogel in steam sensing as shown in Figure S15, Note S3, and Table S3 (Supporting Information).

## 3 Conclusion

In summary, a terpolymer ionogel combined with FEP as the tribonegative layer and Al as the top electrode formed a three‐layer i‐REH. The ionogel exhibits high transparency, exceeding 92% in the visible region. Moreover, it demonstrates strong adhesion, with a maximum shear stress of 0.29 MPa (FEP‐ionogel), respectively, which allows for a robust i‐REH structure that is suitable for various application scenarios. Due to the polarization interface between the conductive ionogel and FEP, the charging time is significantly reduced with enhanced instantaneous charge stability and electrical output. Every individual deionized water droplet can generate current, voltage, and charge of up to 165 µA, 175 V, and 22.5 nC. Besides, every individual tap water droplet can generate current and voltage of up to 502 µA and 204 V, with an energy conversion efficiency of 6.77%. Additionally, the device was utilized in steam sensing. When steam leakage occurs, the DC voltage output increases to 23 mV and subsequently returns to its original value over a period of 1000 s. These results not only highlight the potential of i‐REH in energy harvesting but also steam monitoring, paving the way for further research and development in both energy and sensor technologies.

## 4 Experimental Section

### Synthesis of Ionogel

A terpolymer ionogel was prepared through a free‐radical polymerization process. Deionized water was used to dissolve the following components: 55.7 wt.% acrylic acid (99%, Aladdin), 29.1 wt.% acrylamide (99%), and 5.8 wt.% 2‐acrylamido‐2‐methyl‐1‐propanesulfonic acid (98%, Aladdin). A total of 2.33 g monomer was added to 8 mL of deionized water. Anhydrous sodium borate (Macklin) was then added as the crosslinking agent, followed by 15 min of sonication to ensure homogeneity. Next, 20 µL of the accelerator tetramethyl ethylenediamine (99%, Aladdin) was added, and the solution was heated at 70 °C on a hotplate for 2 min. Subsequently, 2 mL of a 5 wt.% aqueous ammonium persulfate (98%, Aladdin) solution was incorporated while stirring at 700 rpm. Finally, the reaction mixture was covered with aluminum film and heated at 70 °C for an additional 20 min to complete the polymerization process.

### Material Characterization

The chemical bonds and functional groups of the ionogel were identified by FTIR (PerkinElmer FTIR Spectrometer) equipped with an attenuated total reflectance (ATR) sampling mode. The spectra were recorded in the range of 500–4000 cm^−1^. The absorbance and transmittance were measured using a UV–vis spectrophotometer (Shimadzu 2600) by applying the ionogel onto a glass substrate with a wavelength range from 280 to 900 nm. The water contact angle of both the ionogel and FEP film was tested with a contact angle tester by putting a 5 µL water droplet on both surfaces of the ionogel and FEP. The ionogel was evenly spread and supported by a glass plate (Data Physics Contact Angle Tester). For adhesion property, both the shear force and shear stress were measured using a mechanical tester (Instron 34SC‐05). The glass substrate and thin films, including FEP, PTFE, PDMS, PET, and nylon, were cut into cuboids with a width of 2 cm and a length of 5 cm. A small piece of ionogel was then applied to adhere two cuboids together in a square area with sides of 2 cm. The contact potential difference was measured using KPFM (Bruker Dimension Icon). The applied HQ: NSC18/Pt probes (Mikromasch, China) operate at a resonance frequency of 75 Hz with a tip diameter of 30 nm.

### Fabrication of Rain Energy Harvester

The ionogel was uniformly applied to a 3 cm × 3 cm glass substrate as the bottom electrode. Next, a commercial FEP film was affixed to the ionogel to function as the tribonegative layer. A small piece of aluminum tape with dimensions of 0.3 cm × 3 cm was then adhered to FEP as the top electrode. Finally, the top and bottom electrodes were connected using an external wire.

### Design and Rain Simulation

A cylindrical plastic bucket was utilized for water storage. The droplet was generated by a syringe pump connected to the bucket, whose velocity was adjusted by rotating a valve. The droplet size was controlled by the inner radius of the nozzle of the plastic tube. Additionally, the droplet height was modified by placing i‐REH in different layers within the droplet simulation setup. At this time, the droplet size, frequency, and height were fixed at 60 µL with an inner radius of 5 mm, 1.2 droplets s^−1^ (equivalent to a flow rate of 4.5 mL min^−1^), and 12 cm.

### Electrical Measurement

The short‐circuit charge was measured by a programmable electrometer (Keithley 6517B). The voltage output was measured using an oscilloscope (RIGOL DS1054). The current output was measured by a low‐noise current preamplifier (Stanford Research System Model SR570) connected to the oscilloscope. The direct‐circuit voltage and current output of the steam sensor and PV cell were measured by a digital multimeter (UNI‐T UT33B+). The irradiation was simulated by a solar simulator (ENLITECH), and the voltage outputs of the PV cell were measured by a digital multimeter (UNI‐T UT33B+).

### Statistical Analysis

Data reported are expressed as ± s.e.m in Figure 3a,b,e. Error bars in all figures represent standard deviation. Sample sizes are 5, 4, and 5. All analyses were performed using Origin 2021.

## Conflict of Interest

The authors declare no conflict of interest.

## Supporting information



Supporting Information

Supplemental Movie 1

Supplemental Movie 2

Supplemental Movie 3

Supplemental Movie 4

## Data Availability

The data that support the findings of this study are available from the corresponding author upon reasonable request.
