# Supplementary material for: Fast Charging and High Power Ionogel‐Based Rain Energy Harvester
Source: Adv Sci (Weinh). 2025 Jun 30;12(35):e04608. doi: 10.1002/advs.202504608 (PMC12462965; doi:10.1002/advs.202504608)
Supplement: Supplementary file 1 — Supporting Information [file ADVS-12-e04608-s003.docx]

**Supporting Information**

**Fast Charging and High Power Ionogel-Based Rain Energy Harvester**

*Ruoxuan Ye^1#^, Irum Firdous ^1#^, Muhammad Fahim^1^, Jihong Shi^1^, Weilu Li^1^, Xiangkun Bo^1^, Fei Lui^1^, Walid A. Daoud^1,2^**

^1^ Department of Mechanical Engineering, City University of Hong Kong, Hong Kong, China

^2^ Shenzhen Research Institute, City University of Hong Kong, Shenzhen, China

* Corresponding author. Email: wdaoud@cityu.edu.hk

^#^ These authors contributed equally to this work.

**
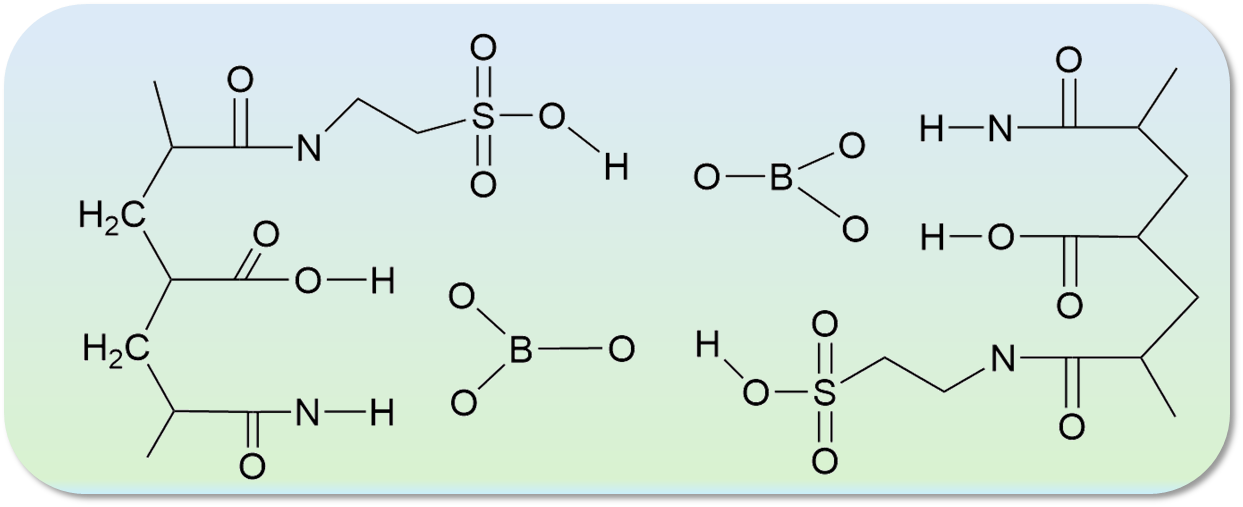
**

**Figure S1.** Chemical composition of the terpolymer. The main chain is composed of three monomers, with each contributing distinct functional groups, where the carboxyl, amide, and sulfate groups originate from AA, Aam, and AMPS, respectively.

**b**

**a**


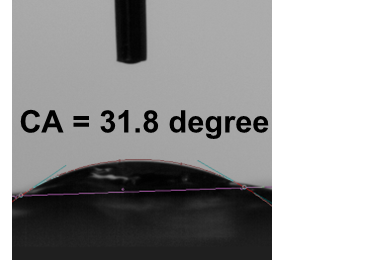

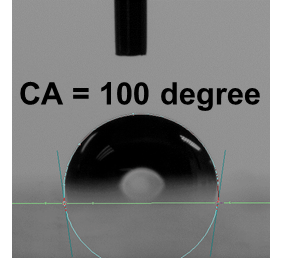


**Figure S2.** The water contact angle (CA). (a) The water droplet on the ionogel. (b) The water droplet on the FEP film.

**
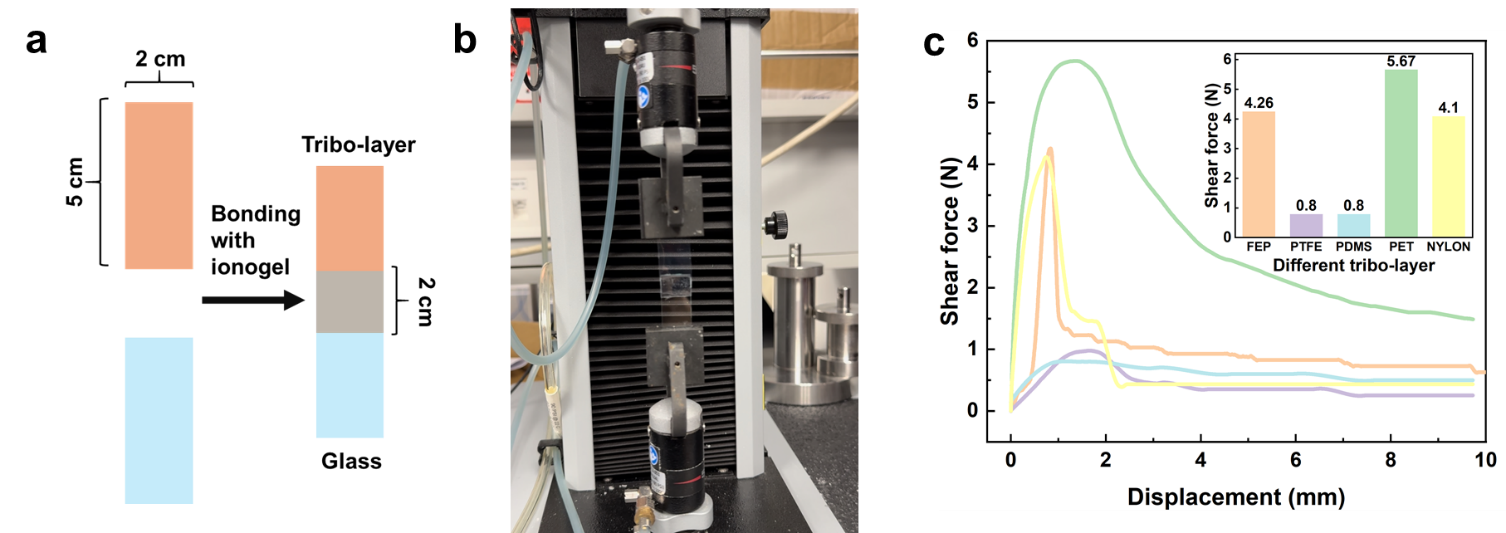
**

**Figure S3.** Adhesion Test. (a) The tribo-layer (orange part) and glass substrate (blue part) were bonded by the ionogel (grey part). Then, they gradually separated by the tester. (b) Photo of tester. (c) Shear force with displacement of five different layers.


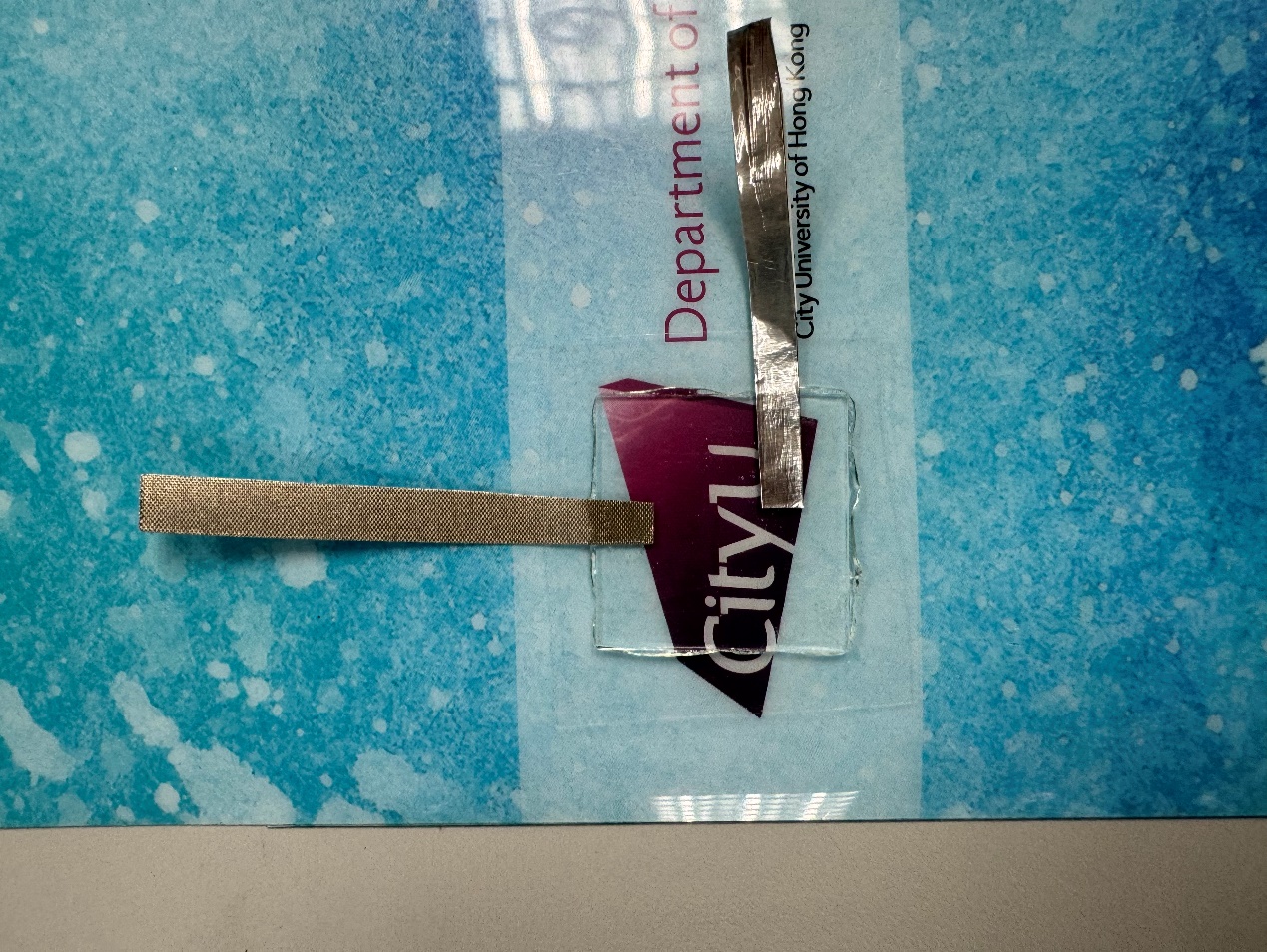


**Figure S4.** Digital photo of i-REH (Components: A top electrode-Aluminum, a tribonegative layer-FEP, a bottom electrode-ionogel, and a glass substrate) with overall high transparency.

**b**

**a**


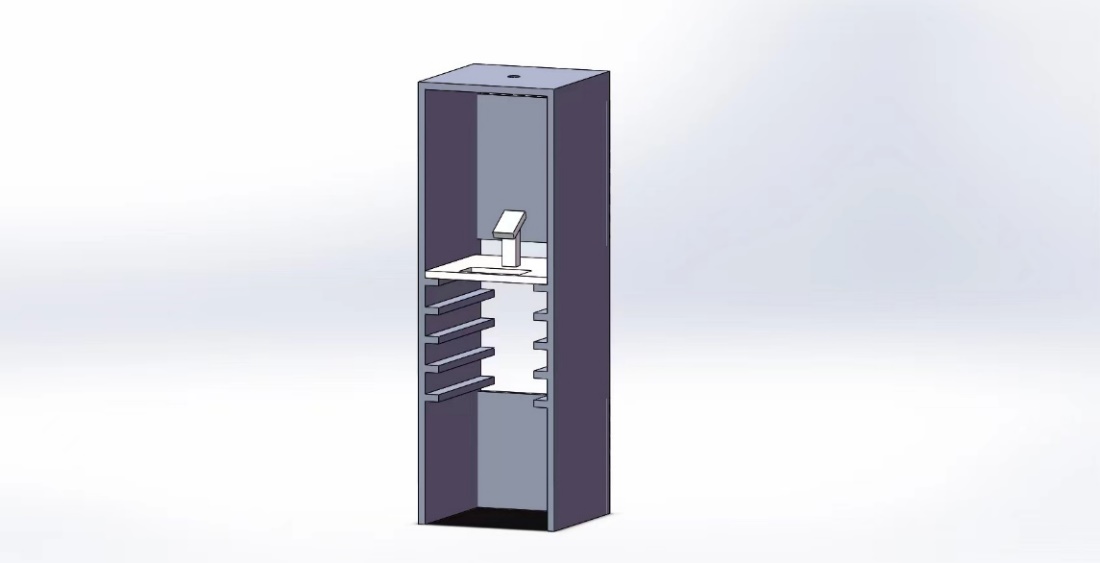

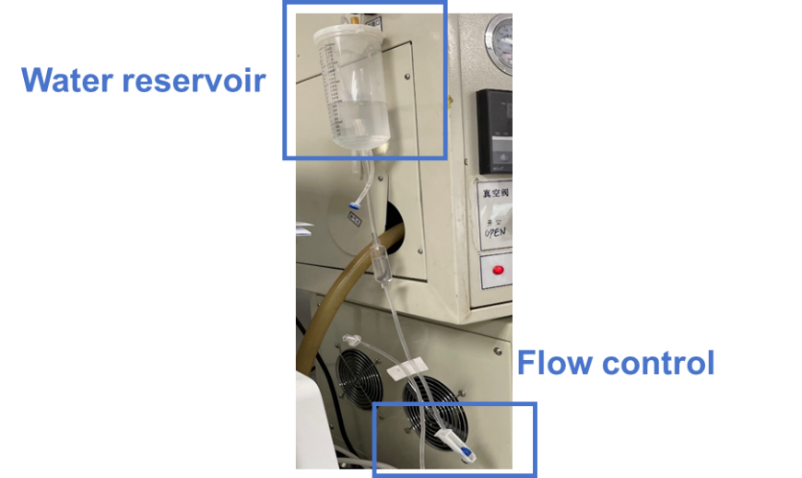


**Figure S5.** Droplet simulation setup. (a) Model design used for droplet height and inclination angle control. (b) Water reservoir and droplet flow velocity control.

**
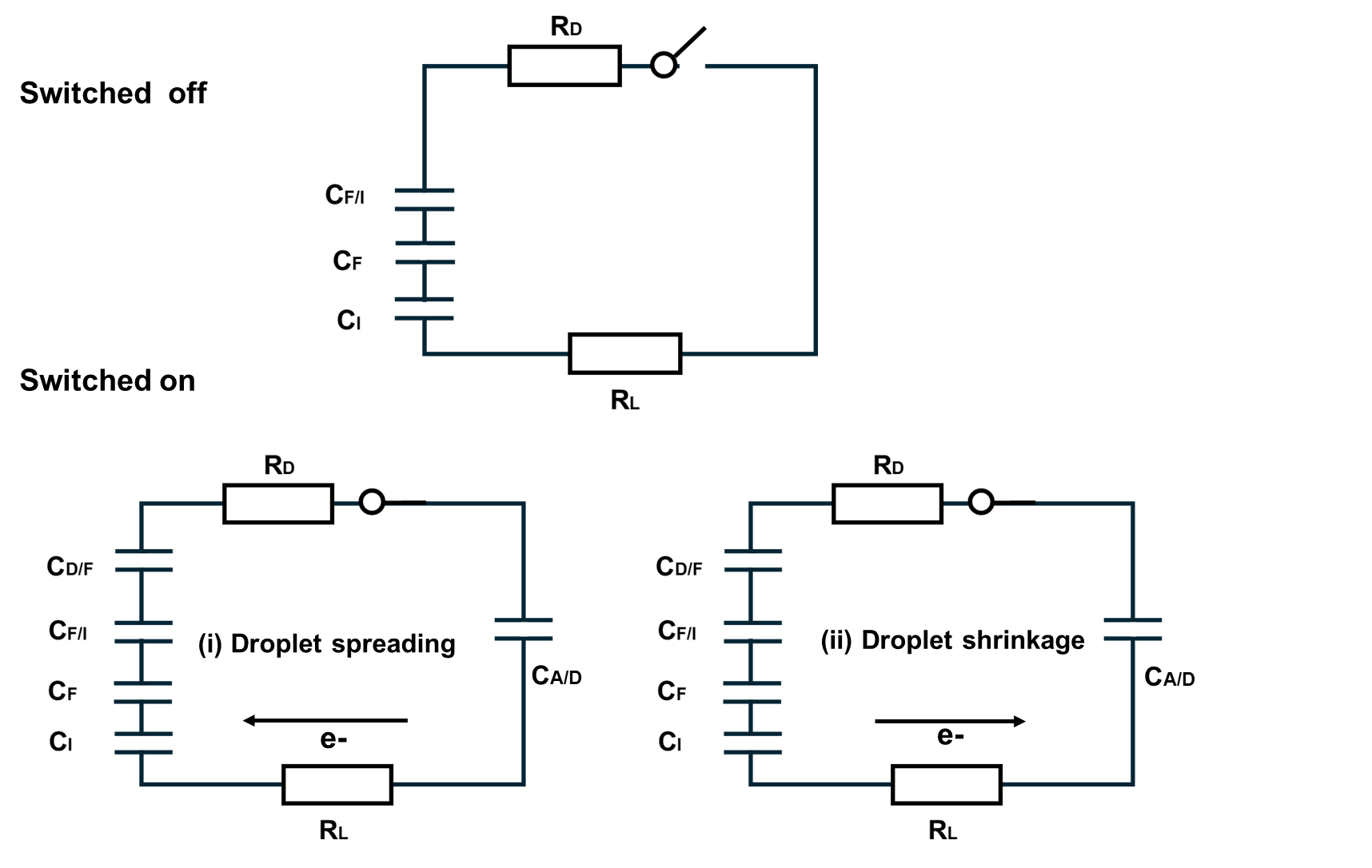
**

**b**

**a**

**Figure S6.** Detailed equivalent circuit diagrams. (a) State of the switch off when droplets are not in contact with i-REH, indicating no charge transfer. (b) State of the switch on: (i) Electrons flow clockwise in the equivalent circuit when droplets spread. (ii) Electron flow counterclockwise in the circuit when droplets make contract.


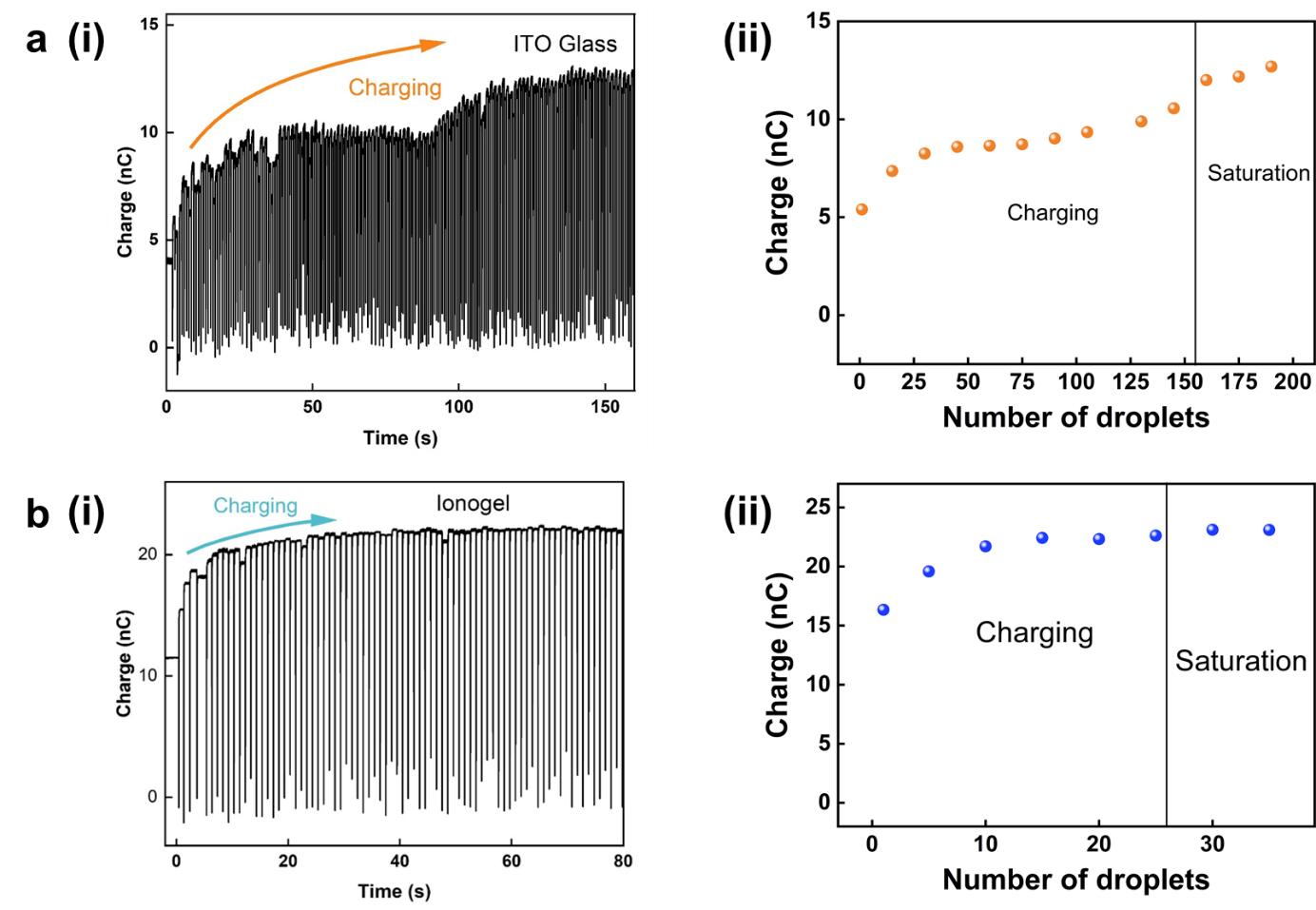


**Figure S7.** Charging process of different droplet energy harvesters (droplet volume and frequency are fixed at 60 μL and 1.2 droplets per second). (a) ITO glass serves as the bottom electrode, which requires over 150 droplets to be saturated: (i) Original charge curve changed with the increase of droplets. (ii) Processed charge values changed with the increase of droplets. (b) Ionogel serves as the bottom electrode, which requires only 25 droplets to reach saturation: (i) Original charge curve changed with the increase of droplets. (ii) Processed charge values changed with the increase of droplets.

**
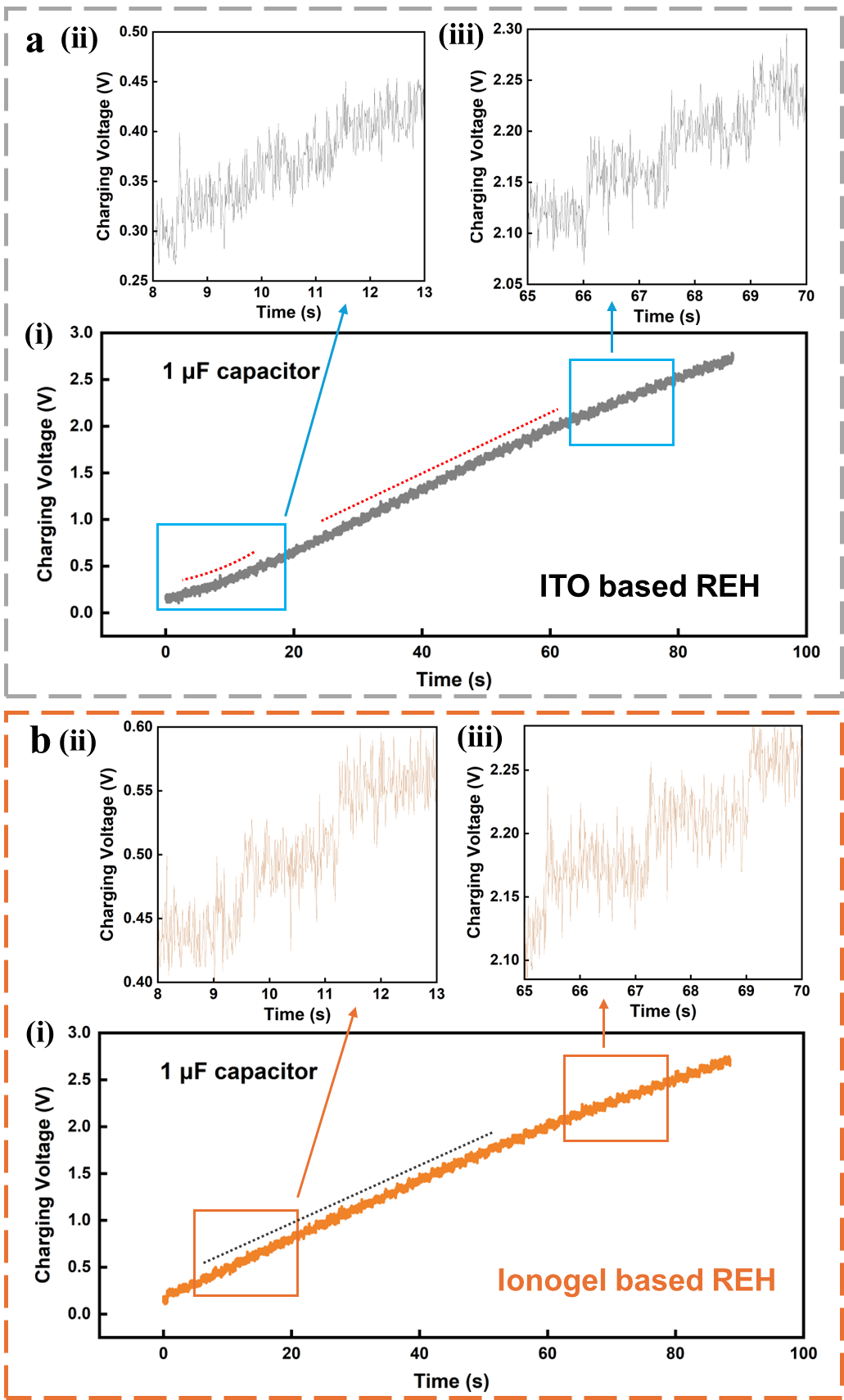
**

**Figure S8. Comparison of the capacitor charging process.** Both (a) ITO-based and (b) Ionogel-based REH are initiated from unsaturated states. The droplet volume and frequency are fixed at 60 μL and 0.6 droplets per second. The capacitor charging process for ITO-based REH (a(i)) corresponds to Figure S7(a). It requires a greater number of droplets to achieve maximum output, resulting in a slower initial capacitor charging speed (a(ii)), which then becomes constant (a(iii)). Conversely, the capacitor charging process for i-REH (b(i)) is stable, maintaining a constant capacitor charging speed in both the beginning (b(ii)) and the end state (b(iii)). (For this experiment, the droplet height was adjusted to ensure similar voltage output at saturation for both ITO and ionogel-based REHs.)

**
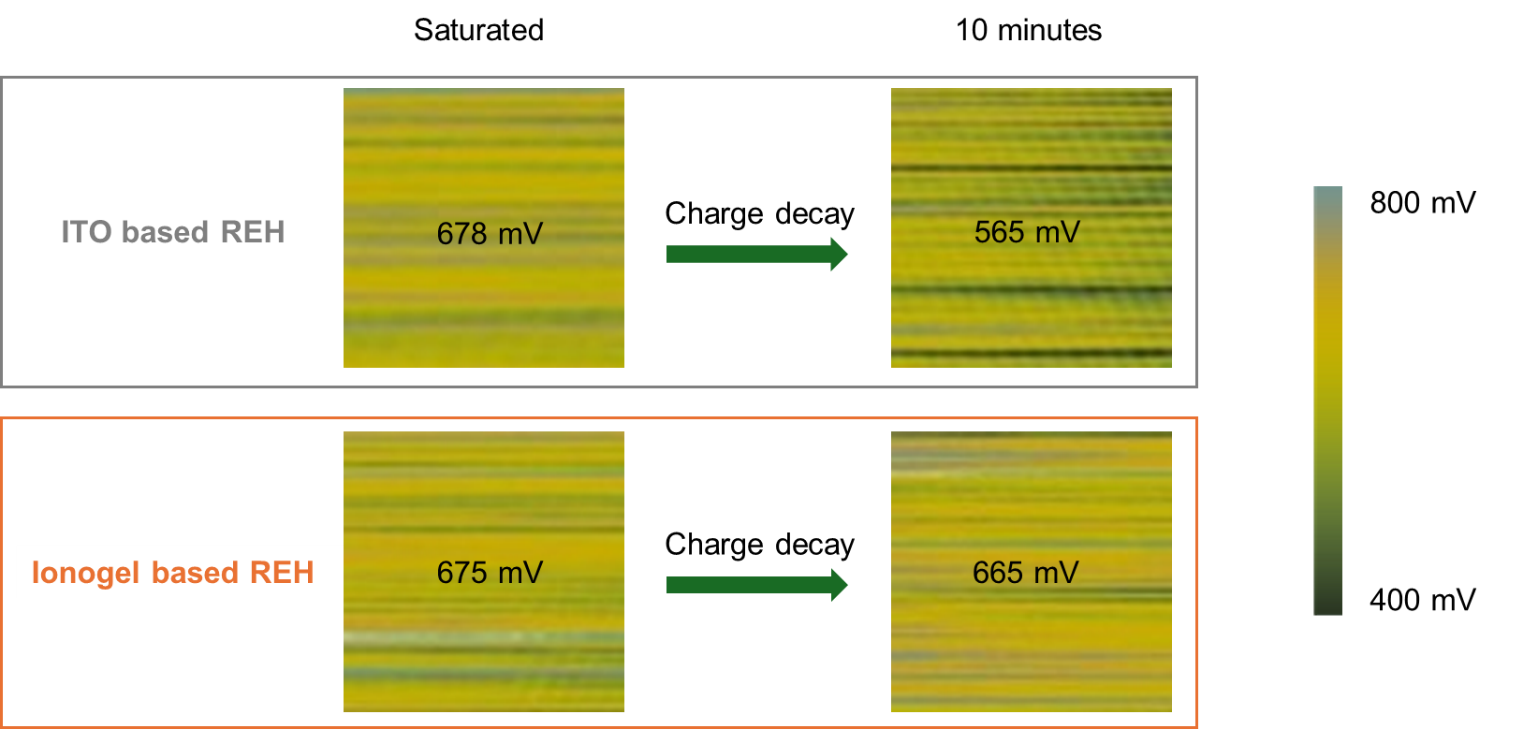
**

**Figure S9.** Surface charge distribution and decay over time using KPFM. The contact potential difference (CPD) is defined as the potential of the tip minus the potential of the sample. Both ITO-based REH and ionogel-based REH were brought into contact with aluminum 150 times to fully saturate their surface for the initial mapping. To illustrate the charge decay rate, both saturated REHs were left in the air for 10 minutes before the second mapping. Due to the presence of the same tribonegative layer, they exhibited very similar initial CPD of 678 and 675 mV, respectively. However, after 10 minutes, the CPD of ITO-based REH decreased to 565 mV. While ionogel-based REH, influenced by the inherently charged layer caused by the closely fitted ionogel and FEP layers, showed a significantly smaller charge decay with only a 10-mV reduction in CPD.


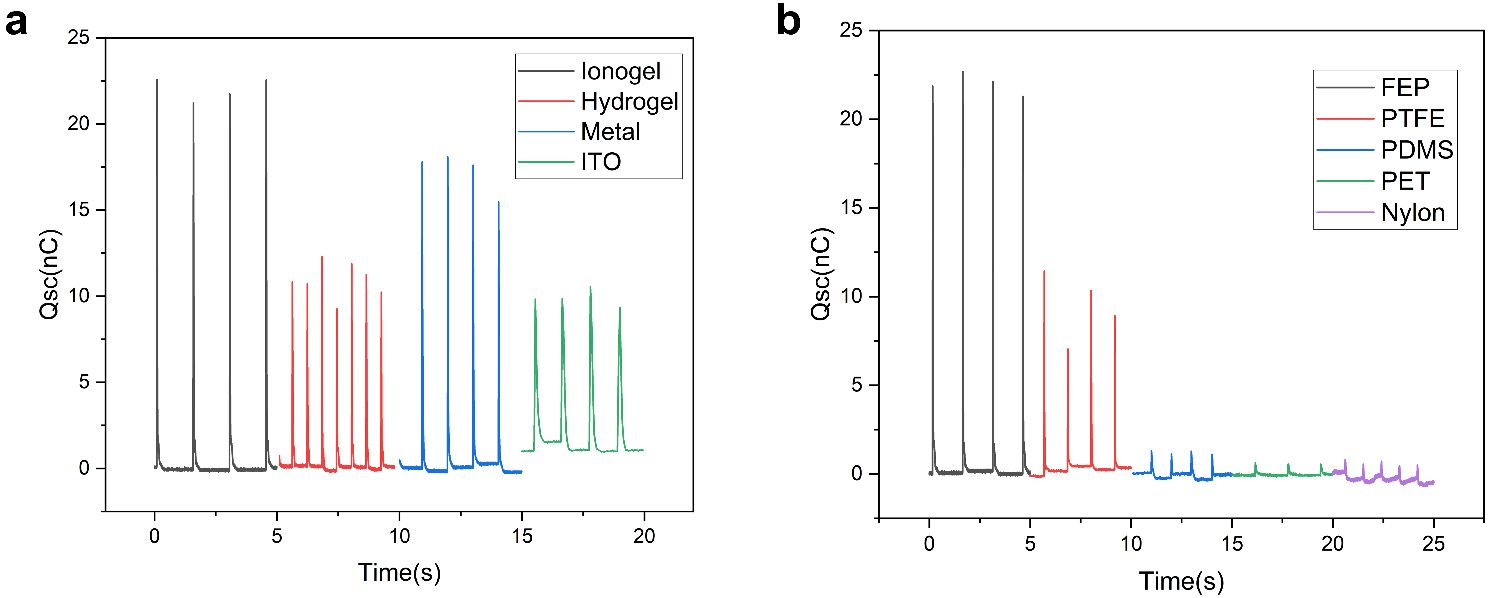


**Figure S10.** Comparisons of different bottom electrodes and tribo-layer materials. (a) Short Circuit charge (Q_sc_) outputs of different bottom electrodes, ionogel, hydrogel^[1]^, metal tape, and ITO glass. (b) Short circuit charge outputs of different tribo-layers, FEP, PTFE, PDMS, PET, and nylon.


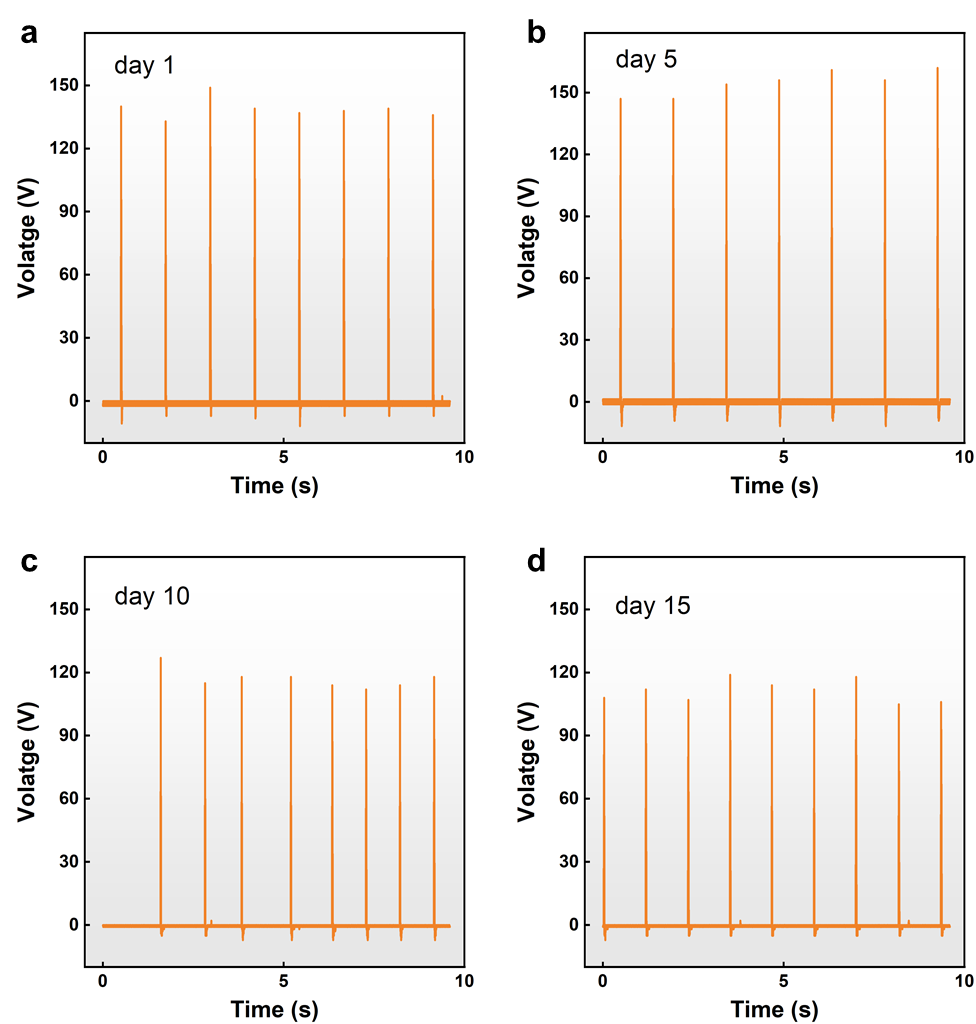


**Figure S11.** Durability of i-REH. (a) Day 1. (b) Day 5. (c) Day 10. (d) Day 15.


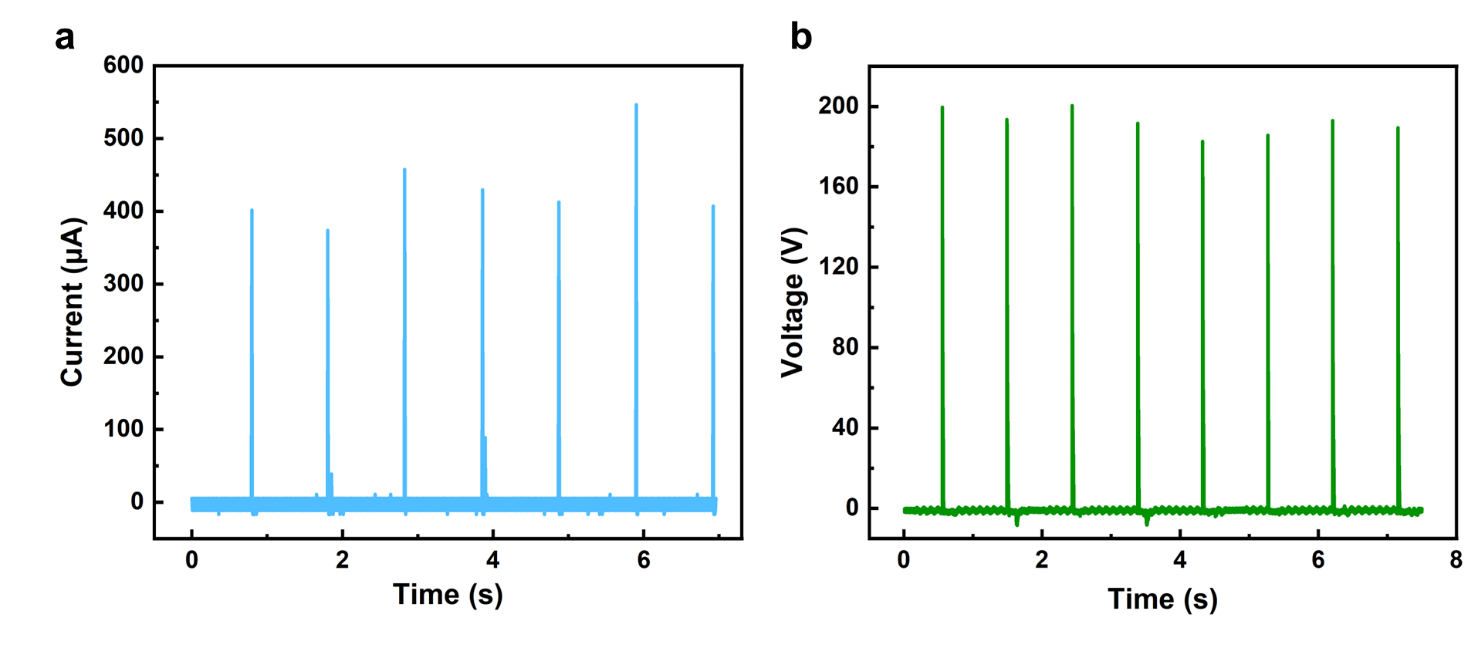


**Figure S12.** Electrical outputs from tap water droplets. (a) The maximum current output is ≈ 502 μA. (b) The maximum voltage output is ≈ 204 V.


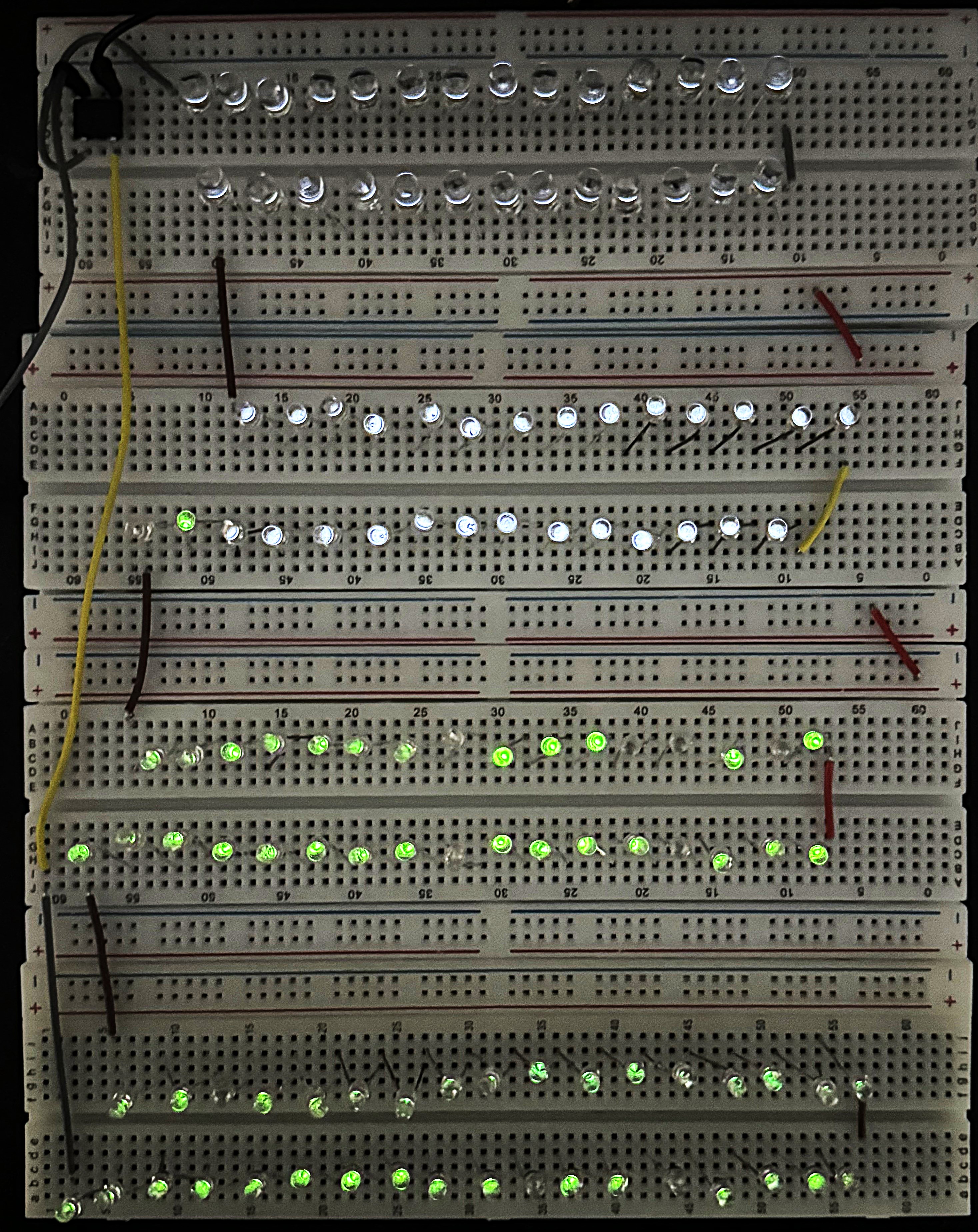


**Figure S13.** LEDs illumination.


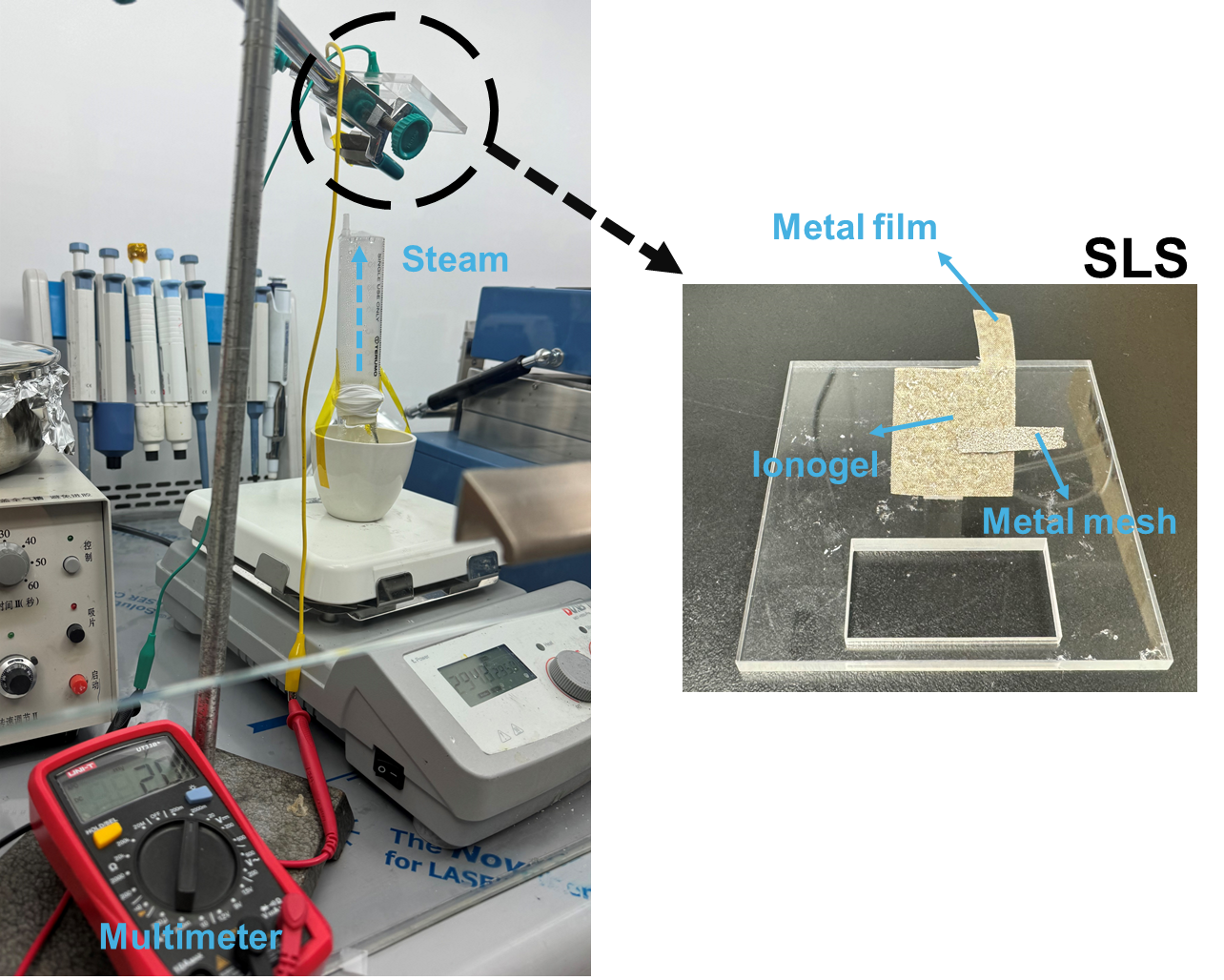


**Figure S14.** Digital photos of steam leakage sensor and the steam simulation setup. DI water is boiled on a hot plate. Generated steam flows out from the tip of the needle. SLS detects steam and generates an electrical signal as recorded on a digital multimeter.

**
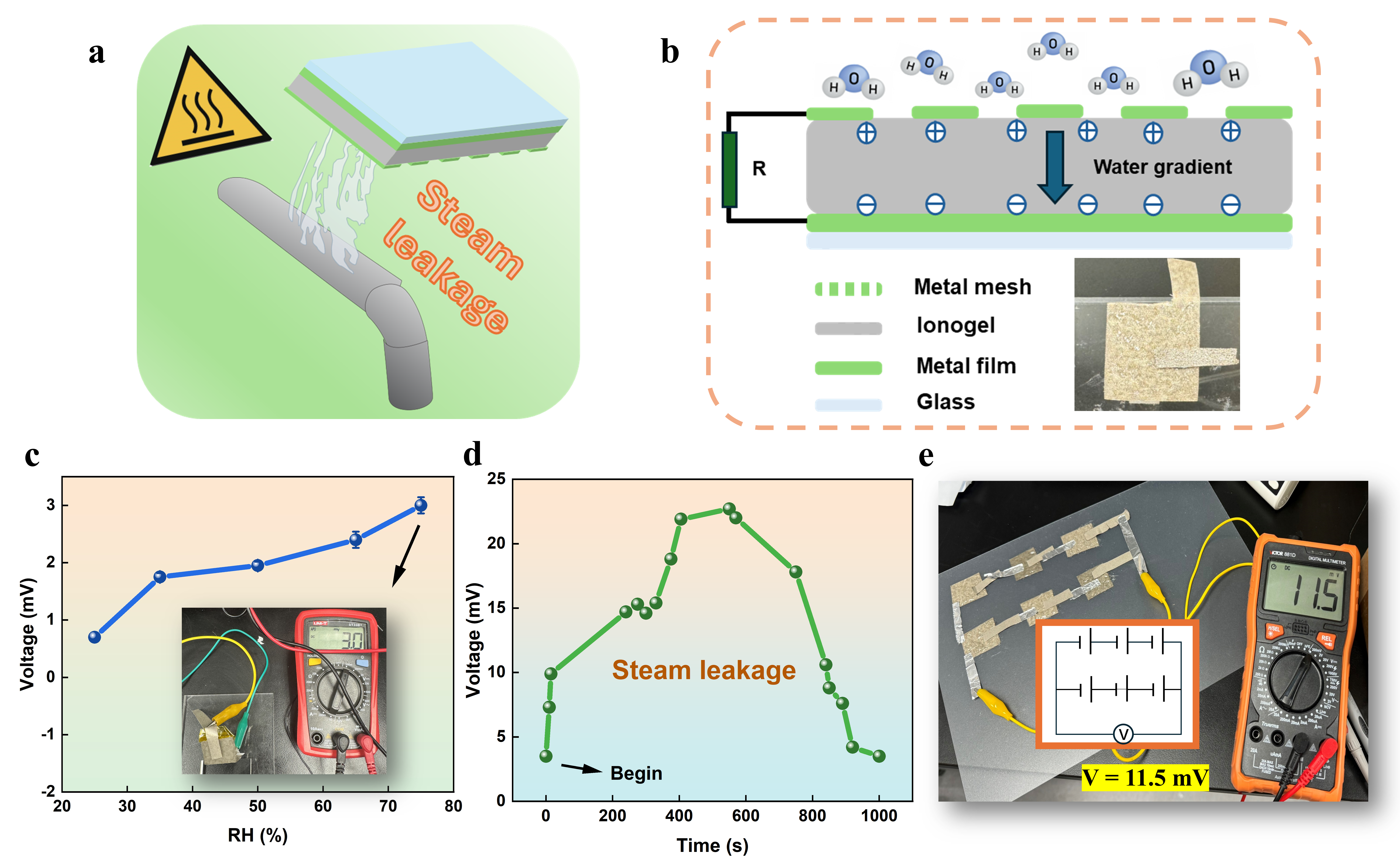
**

**Figure S15. Application in steam leakage sensing (SLS).** (a) Schematic diagram of SLS’s working scenario. (b) Structure and working mechanism of SLS. (c) Direct-circuit (DC) voltage output with increasing relative humidity (RH). Data reported are means ± s.e.m. For each mean, the total number for measurement is 4. (d) DC voltage increases with steam leakage, followed by a recovery process. (e) DC voltage and current of an array of SLSs.


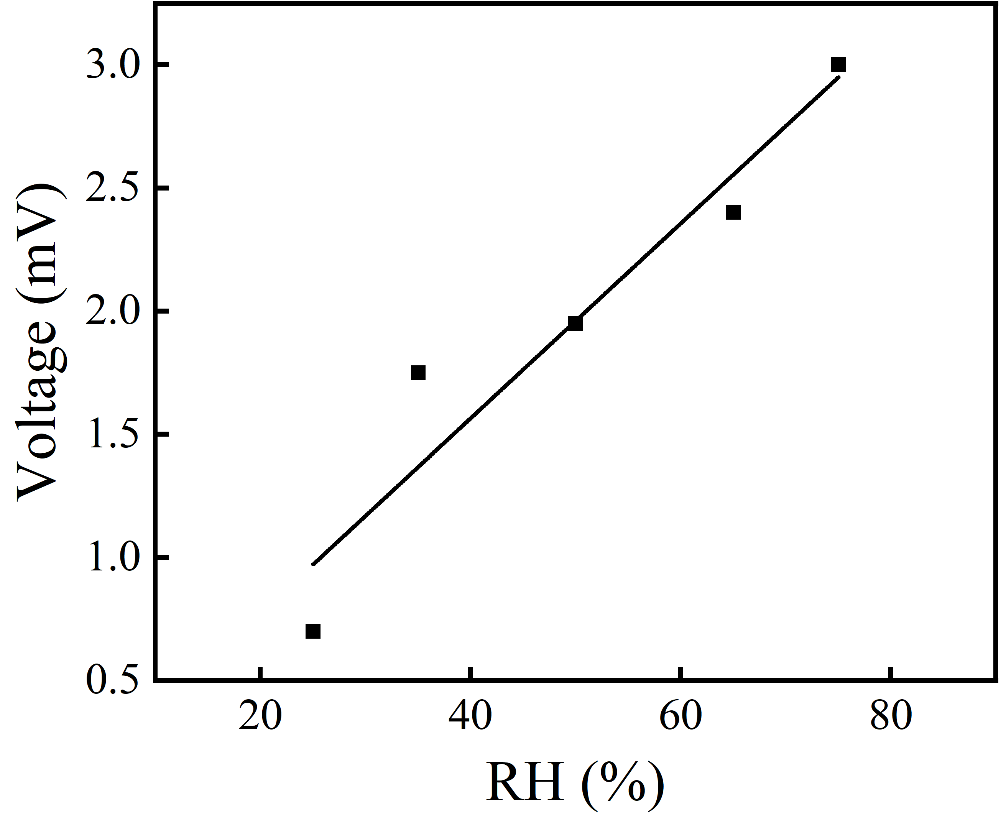


**Figure S16.** The fitted curve is represented by the equation: y=0.03956x-0.01794, where x is the environmental relative humidity and y is the voltage output from the sensor. The intercept of -0.01794 indicates that when humidity is zero, the predicted output of SLS is close to zero, indicating a reasonable output. A slope of 0.03956 means that for every 1% increase in humidity, voltage output also increases commensurably. The calculated coefficient of determination, R^2^, is 0.91515, which is close to 1, indicating a linear response.


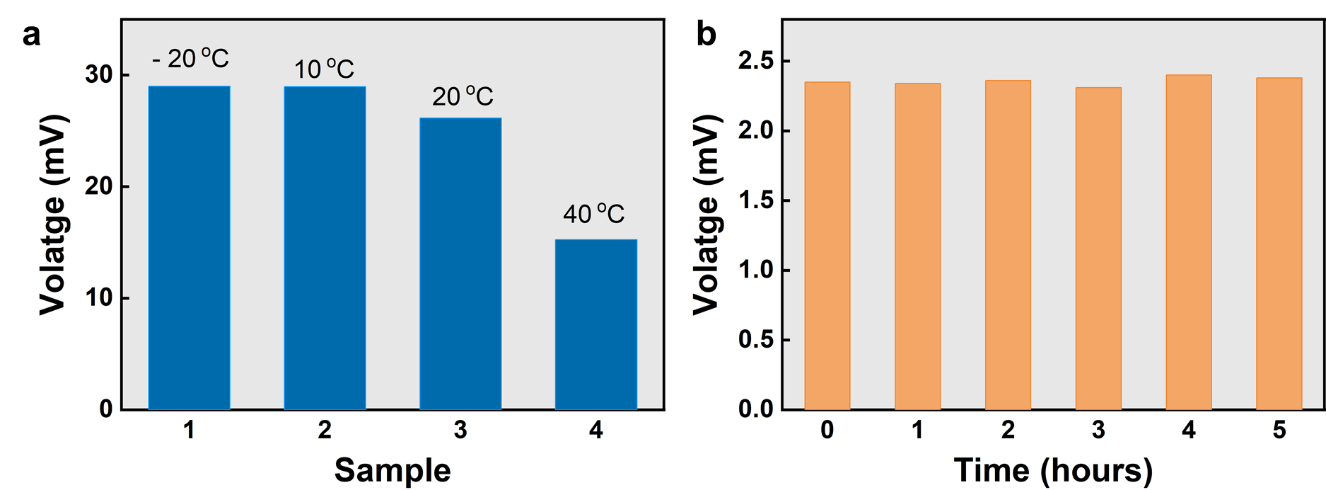


**Figure S17.** Performance of SLS. (a) at different temperatures. (b) at ambient temperature and humidity (23 ℃ and 65%) for 5 h.

**Supplementary Note S1: Detailed analysis of UV-Vis and FTIR spectra.**

The FTIR analysis of the terpolymer ionogel, as illustrated in **Figure 1b**, reveals distinctive peaks within specific frequency ranges. Notably, signals observed between 3350 and 3200 cm^-1^ correspond to the stretching vibration of hydroxyl and amido groups derived from the AA and Aam molecules.^[2, 3]^ A prominent peak at 2960 cm^-1^ signifies the -CH_2_- vibration, corroborating the successful polymerization of the terpolymer.^[4]^ Further spectral analysis unveils stretching vibrations at 1455 and 1655 cm^-1^, indicative of C=O bonds, encompassing characteristic features of the constituent monomers, including C-O, C-C, and O=C-C bonds. This amalgamation underscores the pronounced vibration of C=O, affirming the synthesis of the targeted materials, AA and Aam.^[5]^ Moreover, discernible features include a C-H bending band at 1420 cm^-1^ and a S=O stretching band at 1048 cm^-1^.^[6]^ Notably, the absence of the olefinic C=C band confirms the successful formation of a copolymer through successful synthesis.^[7]^

As shown in the UV-Vis spectrogram in **Figure 1c**, a distinct absorption peak is observed in the range of 300 to 400 nm for this terpolymer. This absorption primarily originates from the AA unit, where the carboxyl group in the AA molecule facilitates a $\pi-\pi^{*}$ jump through the conjugation effect, resulting in relatively strong light absorption.^[8, 9]^ In contrast, the Aam molecule lacks a conjugated structure such as C=C, and the electron transitions are predominantly concentrated in the deep UV region below 200 nm, which does not exhibit significant absorption in the 300-400 nm range.^[10, 11]^ The primary absorption of AMPS occurs at 200-250 nm, corresponding to the amide group transition.^[12]^ Thus, the UV-Vis spectrum of the terpolymer underscores the crucial role of AA in the copolymerization process. Furthermore, the overall transmittance in the visible range of 400-700 nm is approximately 92%, indicating that this ionogel is highly transparent, and the logo of CityU can be observed. The high transparency offers numerous practical applications, such as direct assembly on a PV cell to achieve multiple energy harvesting.

**Supplementary Note S2: Calculation of energy conversion efficiency.**

To examine the working efficiency of i-REH, the conversion efficiency of droplet mechanical energy to electrical energy generated is calculated using **Equation S1**:

$\eta_{REH}=\frac{E_{electricity}}{E_{droplet}}=\frac{\int U^{2}/Rdt}{mgh}$ (S1)

Where electrical energy generated by one tap water droplet is 4.78 x 10^-6^ J, through calculating the integral of the square of voltage over time, which is divided by the external resistance, 5 MΩ (**Figure S18**). The mass, m, of one droplet is calculated by $m=\rho V$, where $\rho$ is the water density, 1 gml^-1^, V is the volume of droplet, 60 μL, g is the gravitational acceleration, 9.8 ms-^2^, and h is the height of droplet, 12 cm. Hence, the calculated mechanical energy from one droplet is 7.06 x 10^-5^ J, with an overall device energy conversion of 6.77%.


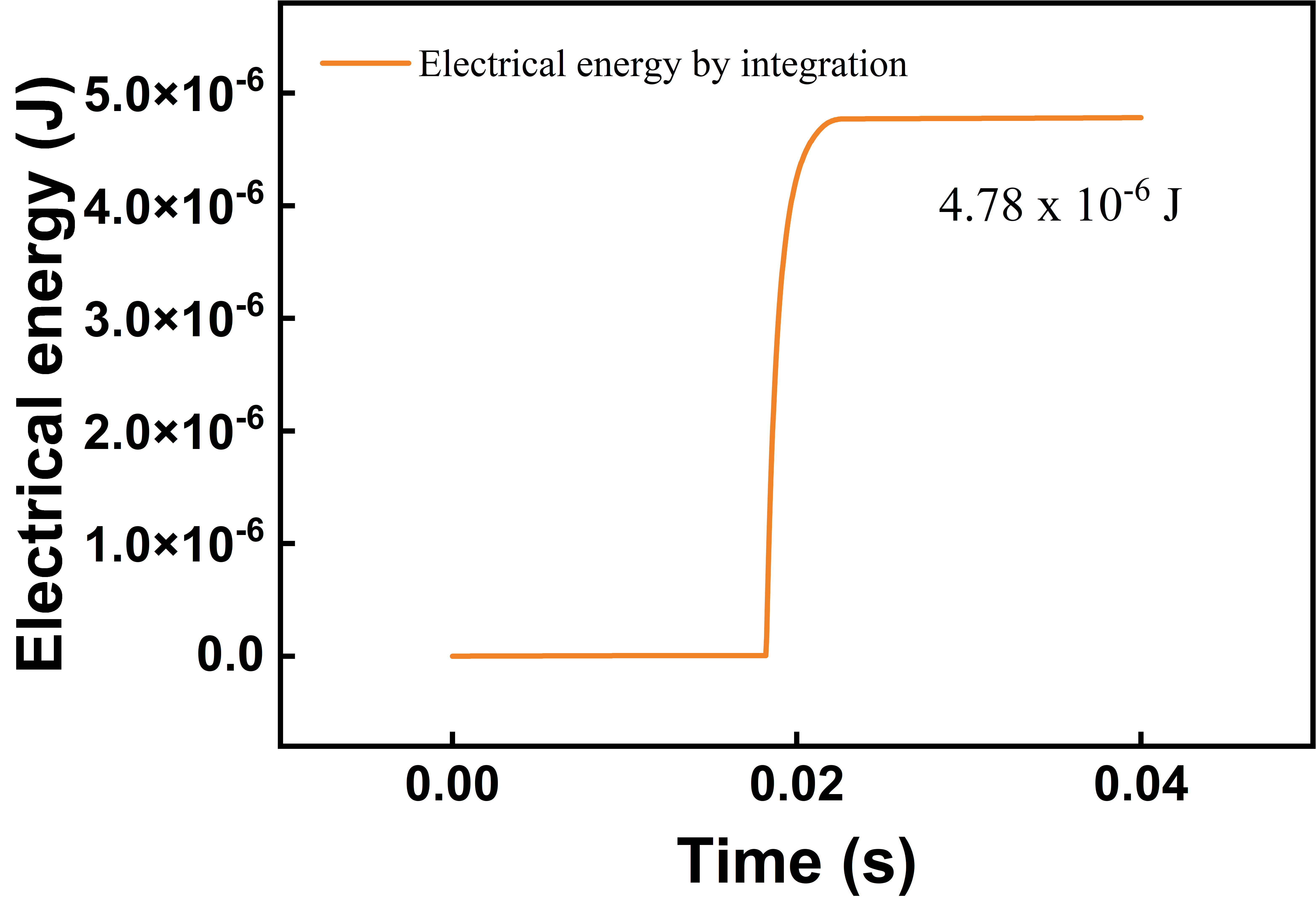


**Figure S18.** The amount of electrical energy generated from a single droplet impinging on i-REH. The integration of U^2^R^-1^ is calculated by Origin 2021.

**Supplementary Note S3: Steam leakage sensing.**

Leveraging the unique characteristics of the ionogel and the sandwich structure of i-REH, the device’s capability to generate electricity upon contact with steam was demonstrated. To be more specific, the device can function as a steam leakage sensor, where the generated electrical signal upon steam detection serves as a warning alarm alerting nearby individuals. Digital photos of SLS and the steam simulation platform are shown in **Figure S14**. This is crucial as steam can pose significant safety hazards. For instance, SLS can be employed to monitor steam leakage of industrial equipment, such as refrigeration units and steam pipelines, where leakage could lead to operational inefficiency and equipment damage, as shown in **Figure S15a**.

The working mechanism of SLS is similar to that of a moisture electricity generator (MEG), where electricity is generated through the migration of ions in water vapor, driven by molecular flow or concentration gradient resulting from the non-equilibrium release of ions upon moisture adsorption onto a solid surface. MEG effectively converts the chemical energy contained in moisture into electrical energy, only by utilizing a straightforward structure composed of readily available materials.^[13]^

The hydrophilic properties of the ionogel are crucial to the functionality of MEG. Hydrophilicity enhances the absorption and retention of vapor, which is vital for establishing moisture gradients. The compositions of AA and AMPS exhibit hydrophilic characteristics that facilitate moisture absorption, as shown in **Figure S2a**. Moreover, the porous nature of the ionogel allows for effective water permeation via the capillary effect.

The presence of various ions, such as H^+^ and OH^-^, within the hydrophilic ionogel contributes to the establishment of ion concentration gradients. Following the dissociation of water molecules, these ions hydrate the ionic liquid, generating ionic charge carriers. Notably, AMPS molecules contain strong acidic groups -SO_3_H, that can completely dissociate into sulfate ions.^[14, 15]^ Upon water absorption, the deformation of the polymer matrix promotes the relatively free migration of sulfate ions, leading to the formation of a spontaneous ion gradient that corresponds to the moisture gradient across the film thickness. This gradient drives the directional migration and transmembrane transport of ions along the polymer chains.^[16, 17]^

SLS comprises a four-layer structure, as illustrated in **Figure S15b**. A mesh layer of conductive metal covers the upper surface of the ionogel to facilitate steam absorption and enhance access to the ionogel.^[18]^ Beneath the ionogel, a conductive metal film is positioned, supported by a glass plate. This glass plate not only serves as a substrate to support the entire structure, but also seals the lower surface of the ionogel, preventing direct exposure to steam. Consequently, in high-humidity environments or during steam leakage, a moisture and ion concentration gradient develops between the two electrodes, prompting ionic movement that generates an electrical signal.^[19, 20]^

SLS exhibits the fundamental characteristics of a MEG, which establishes a moisture gradient and produces electricity in stable humidity environments. As depicted in **Figure S15c**, output increases with relative humidity (RH), specifically, the DC voltage increases from 0.7 to 3 mV as the RH rises from 25 to 80% (The linearity analysis of SLS is shown in **Figure S16**.). When the sensor is directly exposed to steam (approaching 100% RH), a significant DC voltage increase occurs, reaching 23 mV within 550 s due to direct water vapor absorption. After 980 s, the output stabilizes at its original value, reflecting the dynamics of water absorption and dissipation (**Figure S15d**).^[21]^ This rapid output increase can be used as an indicator of steam leakage, serving as a warning alert in industrial and residential settings. The performance of SLS under different temperatures and durability is shown in **Figure S17**.

Furthermore, multiple SLS units can be interconnected in series and parallel configurations to enhance output. As shown in **Figure S15e**, three SLS units are connected in series, with two rows arranged in parallel. In high humidity environments (RH = 80%), the DC voltage is stable at 11.5 mV.

**Table S1:** Comparison with prominent prior works.

| Journal | Voltage  (V) | Current  (μA) | Power density (W/m^2^) | Conversion efficiency  (%) | Droplet size  (µL) | Droplet height  (cm) | Droplet type | Optimal inclination angle  (^o^) | Reference |
| --- | --- | --- | --- | --- | --- | --- | --- | --- | --- |
| Nature Communication | 103.2 | -- | -- | -- | 96 | 20 | DI | 45 | [22] |
| Advanced Material | 103 | 80 | 48.89 | 3.2 | 72 | 16 | Tap | 30 | [23] |
| Nature | 143.5 | 213.7 | 50.1 | 2.2 | 100 | 15 | Tap | - | [24] |
| Advanced Energy Material | 192 | -- | 65 | 3.6 | 60 | 20 | Tap | 30 | [25] |
| The Innovation | 100 | 1500 | 118 | 0.4 | 50 | 12 | Salt | - | [26] |
| Energy & Environmental Science | 266.6 | 273.6 | 83.02 | -- | 53 | 20 | Tap | 30 | [27] |
|  | **174** | **165** | **--** | **--** | **60** | **12** | **DI** | **30** | **This**  **work** |
|  | **204** | **502** | **235.11** | **6.77** | **60** | **12** | **Tap** |  |  |

**Table S2:** Comparison with previous ionogel/hydrogel-based works.

| Journal | Voltage  (V) | Current  (μA) | Power | Droplet | Optimal inclination angle (^o^) | Reference |  |
| --- | --- | --- | --- | --- | --- | --- | --- |
| Small | 55.4 | 14.6 | 688 μW | Rainwater | - | [28] |  |
| Advanced Functional Material | ~45 | 15 | 42 μW | DI water | - | [29] |  |
| **This work** | **174** | **165** | **-** | **DI water** | **30** |  | |
|  | **204** | **502** | **42.32 mW** | **Tap water** |  |  |  |

**Table S3.** Comparison of ionogel properties in steam leakage sensing (SLS) vs. rain energy harvesting (REH).

| **Property** | **Role in i-REH** | **Role in SLS** |
| --- | --- | --- |
| Sulfate-rich network | Enables anion migration to FEP | Facilitates moisture-driven ion gradients |
| Adhesion (0.29 MPa) | Stabilizes FEP-ionogel interface | Ensure sensor durability |
| Transparency (>92%) | Allows solar panel integration | Irrelevant (opaque metal mesh) |
| Ionic conductivity | Supports EDL formation | Drives steam-induced current |

**Other supporting materials**

**Movie S1.**

Demonstration of the rain droplet simulation setup and working condition of i-REH.

**Movie S2.**

The shear strength test between glass and FEP film adhered by the ionogel.

**Movie S3.**

Demonstration of the steam simulation setup and working condition of SLS.

**Movie S4.**

Demonstration of powering the stopwatch.

.

**References**

[1] I. Firdous, M. Fahim, F. Mushtaq, W. A. Daoud, Electrostatically triggered autonomous self-healable and mechanically robust hydrogel in harsh environments for wearable electronics. *Nano Energy* **2023**, *116*, 108817.

[2] G. Sennakesavan, M. Mostakhdemin, L. K. Dkhar, A. Seyfoddin, S. J. Fatihhi, Acrylic acid/acrylamide based hydrogels and its properties-A review. *Polym. Degrad. Stab.* **2020**, *180*, 109308.

[3] L. Feng, H. Yang, X. Dong, H. Lei, D. Chen, pH‐sensitive polymeric particles as smart carriers for rebar inhibitors delivery in alkaline condition. *J. of Appl. Polym Sci.* **2018**, *135*, 45886.

[4] A.-S. A. Bakr, H. Al-Shafey, E. I. Arafa, A. M. El Naggar, Synthesis and characterization of polymerized acrylamide coupled with acrylamido-2-Methyl-1-propane sulfonic acid-montmorillonite structure as a novel nanocomposite for Cd (II) removal from aqueous solutions. *J. Chem. Eng. Data* **2020**, *65*, 4079.

[5] Y.-M. Wu, B.-Q. Zhang, T. Wu, C.-G. Zhang, Properties of the forpolymer of N-vinylpyrrolidone with itaconic acid, acrylamide and 2-acrylamido-2-methyl-1-propane sulfonic acid as a fluid-loss reducer for drilling fluid at high temperatures. *Colloid Polym. Sci.* **2001**, *279*, 836.

[6] J. Travas‐Sejdic, A. Easteal, Study of free‐radical copolymerization of acrylamide with 2‐acrylamido‐2‐methyl‐1‐propane sulphonic acid. *J. Appl. Polym. Sci.* **2000**, *75*, 619.

[7] B. Peng, S. Peng, B. Long, Y. Miao, W. Y. Guo, Properties of high‐temperature‐resistant drilling fluids incorporating acrylamide/(acrylic acid)/(2‐acrylamido‐2‐methyl‐1‐propane sulfonic acid) terpolymer and aluminum citrate as filtration control agents. *J. Vinyl. Addit. Technol.* **2010**, *16*, 84.

[8] M. Todica, T. Stefan, S. Simon, I. Balasz, L. Daraban, UV-Vis and XRD investigation of graphite-doped poly (acrylic) acid membranes. *Turk. J. Phys.* **2014**, *38*, 261.

[9] B. Li, X. Lu, Y. Ma, Z. Chen, Thermo-and pH-responsive behaviors of aqueous poly (acrylic acid)/poly (4-vinylpyridine) complex material characterized by ATR-FTIR and UV–Vis spectroscopy. *Eur. Polym. J.* **2014**, *60*, 255.

[10] J. Yang, J. Gao, X. Wang, S. Mei, R. Zhao, C. Hao, Y. Wu, X. Zhai, Y. Liu, Polyacrylamide hydrogel as a template in situ synthesis of CdS nanoparticles with high photocatalytic activity and photostability. *J. Nanopart. Res.* **2017**, *19*, 1.

[11] I. Elashmawi, A. Al-Muntaser, Influence of Co_3_O_4_ nanoparticles on the optical, and electrical properties of CMC/PAM polymer: combined FTIR/DFT study. *J. Inorg. Organomet. Polym. Mater.* **2021**, *31*, 2682.

[12] H. Muhammadi, M. Ghorbanloo, M. Mori, H. Yahiro, Ionic liquid hydrogels based on poly (2-acrylamido-2-methyl-1-propanesulfonic Acid-co-1-vinylimidazole): a Green and efficient catalyst carrier for Ag nanoparticles in oxidation and adsorption of benzyl alcohol in water. *Catal. Lett.* **2023**, *153*, 1635.

[13] D. Shen, W. W. Duley, P. Peng, M. Xiao, J. Feng, L. Liu, G. Zou, Y. N. Zhou, Moisture‐enabled electricity generation: from physics and materials to self‐powered applications. *Adv. Mater.* **2020**, *32*, 2003722.

[14] S. Çavuş, Poly (methacrylamide‐co‐2‐acrylamido‐2‐methyl‐1‐propanesulfonic acid) hydrogels: Investigation of pH‐and temperature‐dependent swelling characteristics and their characterization. *J. Polym. Sci. Part B: Polym. Phys.* **2010**, *48*, 2497.

[15] S. Pal, R. Mondal, S. Guha, U. Chatterjee, S. K. Jewrajka, Homogeneous phase crosslinked poly (acrylonitrile-co-2-acrylamido-2-methyl-1-propanesulfonic acid) conetwork cation exchange membranes showing high electrochemical properties and electrodialysis performance. *Polymer* **2019**, *180*, 121680.

[16] X. Li, D. Lv, L. Ai, X. Wang, X. Xu, M. Qiang, G. Huang, X. Yao, Superstrong ionogel enabled by coacervation-induced nanofibril assembly for sustainable moisture energy harvesting. *ACS Nano* **2024**, *18*, 12970.

[17] S. Wang, D. Zhang, J. Zhou, X. He, S. Y. Zheng, J. Yang, Zwitterionic ionogels with water-mediated stiffness transition for shape memory and moisture electric generation. *Nano Energy* **2024**, *120*, 109166.

[18] J. Bai, Y. Huang, H. Wang, T. Guang, Q. Liao, H. Cheng, S. Deng, Q. Li, Z. Shuai, L. Qu, Sunlight‐coordinated high‐performance moisture power in natural conditions. *Adv. Mater.* **2022**, *34*, 2103897.

[19] Q. Li, M. Zhou, Q. Yang, M. Yang, Q. Wu, Z. Zhang, J. Yu, Flexible carbon dots composite paper for electricity generation from water vapor absorption. *J. Mater. Chem. A* **2018**, *6*, 10639.

[20] X. Liu, H. Gao, J. E. Ward, X. Liu, B. Yin, T. Fu, J. Chen, D. R. Lovley, J. Yao, Power generation from ambient humidity using protein nanowires. *Nature* **2020**, *578*, 550.

[21] T. Xu, X. Ding, C. Shao, L. Song, T. Lin, X. Gao, J. Xue, Z. Zhang, L. Qu, Electric power generation through the direct interaction of pristine graphene‐oxide with water molecules. *Small* **2018**, *14*, 1704473.

[22] S. Zhang, M. Chi, J. Mo, T. Liu, Y. Liu, Q. Fu, J. Wang, B. Luo, Y. Qin, S. Wang, Bioinspired asymmetric amphiphilic surface for triboelectric enhanced efficient water harvesting. *Nat. Commun.* **2022**, *13*, 4168.

[23] C. Ye, D. Liu, P. Chen, L. N. Cao, X. Li, T. Jiang, Z. L. Wang, An integrated solar panel with a triboelectric nanogenerator array for synergistic harvesting of raindrop and solar energy. *Adv. Mater.* **2023**, *35*, 2209713.

[24] W. Xu, H. Zheng, Y. Liu, X. Zhou, C. Zhang, Y. Song, X. Deng, M. Leung, Z. Yang, R. X. Xu, A droplet-based electricity generator with high instantaneous power density. *Nature* **2020**, *578*, 392.

[25] H. L. Wang, B. Zhang, T. Chen, W. Mao, Y. Wang, High‐Efficiency Single‐Droplet Energy Harvester for Self‐Sustainable Environmental Intelligent Networks. *Adv. Energy Mater.* **2023**, *13*, 2302858.

[26] Y. Song, W. Xu, Y. Liu, H. Zheng, M. Cui, Y. Zhou, B. Zhang, X. Yan, L. Wang, P. Li, Achieving ultra-stable and superior electricity generation by integrating transistor-like design with lubricant armor. *The Innov.* **2022**, *3(5)*.

[27] X. Xu, P. Li, Y. Ding, W. Xu, S. Liu, Z. Zhang, Z. Wang, Z. Yang, Droplet energy harvesting panel. *Energy Environ. Sci.* **2022**, *15*, 2916.

[28] Y. Wu, T. J. Cuthbert, Y. Luo, P. K. Chu, C. Menon, Cross‐Link‐Dependent Ionogel‐ Based Triboelectric Nanogenerators with Slippery and Antireflective Properties. *Small* **2023**, *19*, 2301381.

[29] S. Jang, S. Lee, S. A. Shah, S. Cho, Y. Ra, G. Lee, Y. Lee, D. Choi, Hydrogel‐Based Droplet Electricity Generators: Intrinsically Stretchable and Transparent for Seamless Integration in Diverse Environments. *Adv. Funct. Mater*. **2025**, *35*, 2411350.
